# Linking Flow-Derived Local Slip Lengths to Force-Derived Effective Slip Lengths in Nanogaps

**DOI:** 10.1021/acs.langmuir.6c03687

**Published:** 2026-10-26

**Authors:** Hidetaka Ozeki, Naoki Azuma, Kenji Fukuzawa, Kento Shimizu, Yuxi Song, Shintaro Itoh, Hedong Zhang

**Affiliations:** † Department of Micro-Nano Mechanical Science and Engineering, 12965 Nagoya University, Furo-cho, Chikusa-ku, Nagoya 464-8603, Japan; ‡ Department of Complex Systems Science, Nagoya University, Furo-cho, Chikusa-ku, Nagoya 464-8601, Japan

## Abstract

A quantitative understanding of interfacial slip is important for determining boundary conditions and evaluating fluid-flow characteristics in nanogaps relevant to fluidic devices and precision micromechanical systems. Interfacial slip has been conventionally evaluated in terms of the slip length derived from squeeze-force measurements, in which the hydrodynamic force generated within the narrowing gap between solid surfaces is compared with a hydrodynamic model assuming a spatially constant slip length. However, the slip length may depend on local flow conditions and may vary spatially within nanogaps. The relationship between such spatially distributed local slip lengths and the force-derived constant slip lengths remains unclear. In this study, we independently measured the squeeze flow and squeeze force within a nanogap formed between two solid substrates in the same experimental system and compared the constant slip length to the spatially distributed local slip lengths. Flow measurements revealed that the local slip length was not constant but depended on the local shear rate at the interface. It was zero at low shear rates, increased linearly in the transition regime, and reached a constant value at high shear rates. Consequently, the slip length varied spatially, depending on the gap height and lateral position. The measured squeeze force was successfully reproduced by calculations incorporating the spatial distribution of the local slip lengths, demonstrating that the macroscopic force response is governed by this localized variation. The force-derived constant slip length was neither the simple average nor the maximum value of the local slip lengths but rather a pressure-weighted effective quantity that reflects the local slip state in high-pressure regions. These findings clarify the physical meaning of the constant slip length obtained from conventional squeeze-force measurements, provide a framework for interpreting slip-length measurements, and demonstrate the importance of quantifying the local slip length to capture actual slip behavior in nanogap flows.

## Graphical abstract

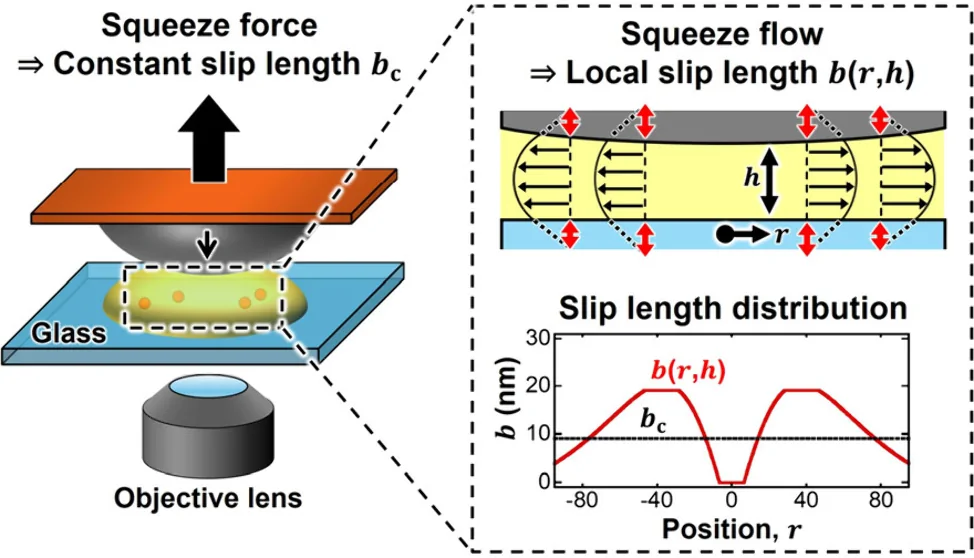

## Introduction

Advances in materials processing technology have improved the smoothness of solid surfaces, and the spaces formed within mechanical systems and devices are rapidly being reduced to the nanometer scale. Fluid flow in such nanospaces is a fundamental phenomenon underlying a wide range of applications, including micro- and nanofluidic devices, thin film lubrication, and micro- and nanoelectromechanical systems.^1–6^ In conventional fluid mechanics, a no-slip boundary condition is typically assumed; however, in nanoscale fluid flows, this condition breaks down, giving rise to interfacial slip, where the fluid exhibits a finite velocity relative to the solid surface.^7–12^ Interfacial slip can occur not only on hydrophobic surfaces but also on hydrophilic surfaces,^13–17^ suggesting that it is a common interfacial phenomenon in nanoscale fluid flows. Since interfacial slip reduces hydrodynamic resistance and pressure loss, a quantitative understanding of the slip behavior is important for improving transport efficiency and reducing friction in fluidic systems.^18–22^

Interfacial slip is commonly characterized by the slip length. The slip length is defined as the distance from the solid surface to the point at which the extrapolated velocity profile reaches zero inside the solid wall, and it represents the flow boundary condition at the solid–liquid interface.^23^ Conventionally, slip lengths have been evaluated through squeeze-force measurements, in which the hydrodynamic force generated during the narrowing of the gap between solid surfaces is compared with a hydrodynamic model assuming a spatially constant slip length.^24–30^ Although this approach has been widely used, the validity of representing slip by a single constant slip length remains unclear for nanogap flows, where local shear conditions vary substantially with the local gap height and lateral position.^31–33^ High-resolution flow measurements and molecular dynamics simulations have shown that the slip length depends on local shear conditions and exhibits a spatial distribution.^16,34–37^ These findings raise an important question regarding the physical meaning of the constant slip length obtained from squeeze-force measurements. Specifically, it remains unclear how the force-derived constant slip length is related to the actual spatial distribution of local slip lengths in nanogaps. Clarifying this relationship is essential for interpreting previously reported slip-length measurements and for establishing physically meaningful boundary conditions in nanoscale fluid flows.

In this study, we independently measured the squeeze flow and squeeze force within a nanogap in the same experimental system to clarify the relationship between spatially distributed local slip lengths and a constant slip length. Local slip lengths were quantified from high-resolution flow measurements using a nanoparticle image velocimetry method developed in our previous study.^38^ The constant slip length was evaluated from squeeze-force measurements using a conventional constant-slip-length model. By quantitatively comparing these results, we clarified how the spatial distribution of the local slip lengths determines the macroscopic force response and the force-derived constant slip length. Furthermore, we elucidated the physical meaning of the constant slip length obtained from the conventional squeeze-force measurements. The findings improve our quantitative understanding of interfacial slip and provide a framework for linking local boundary conditions to macroscopic force responses in nanogap flow.

## Materials and Methods

### Local and Constant Slip Lengths in Squeeze Flow

As shown in Figure 1, when the gap between a spherical substrate and a flat substrate is reduced, the confined fluid is expelled radially outward from the center, generating a squeeze flow. The local gap height at lateral position *r*, *h*(*r*), is expressed as

1 $$h\left(r\right)=h_{0}+\frac{r^{2}}{2R}$$

where *h*_0_ is the minimum gap at the center of the spherical substrate and *R* is the radius of curvature of the spherical substrate. Apparent shear rate *γ̇* at the solid–liquid interface under the no-slip boundary condition in the squeeze flow is expressed as follows:^39^

2 $$\mathrm{\gamma ̇}\left(r,h\left(r\right)\right)=-\frac{3r}{h{\left(r\right)}^{2}}\frac{dh}{dt}$$

where −d*h*/d*t* is the gap-narrowing rate. Equation 2 indicates that the local shear condition varies significantly with lateral position *r* and gap height *h*(*r*), particularly when the gap height reaches the nanoscale. Because interfacial slip is sensitive to local molecular interactions and fluid structuring at the solid–liquid interface, both of which depend on the local shear condition,^8,12,40,41^ the slip length should not be treated as a constant quantity. Instead, it is more appropriately represented by local slip length *b* = *b*(*r*, *h*(*r*)), which depends on *r* and *h*(*r*).

**Figure 1:**
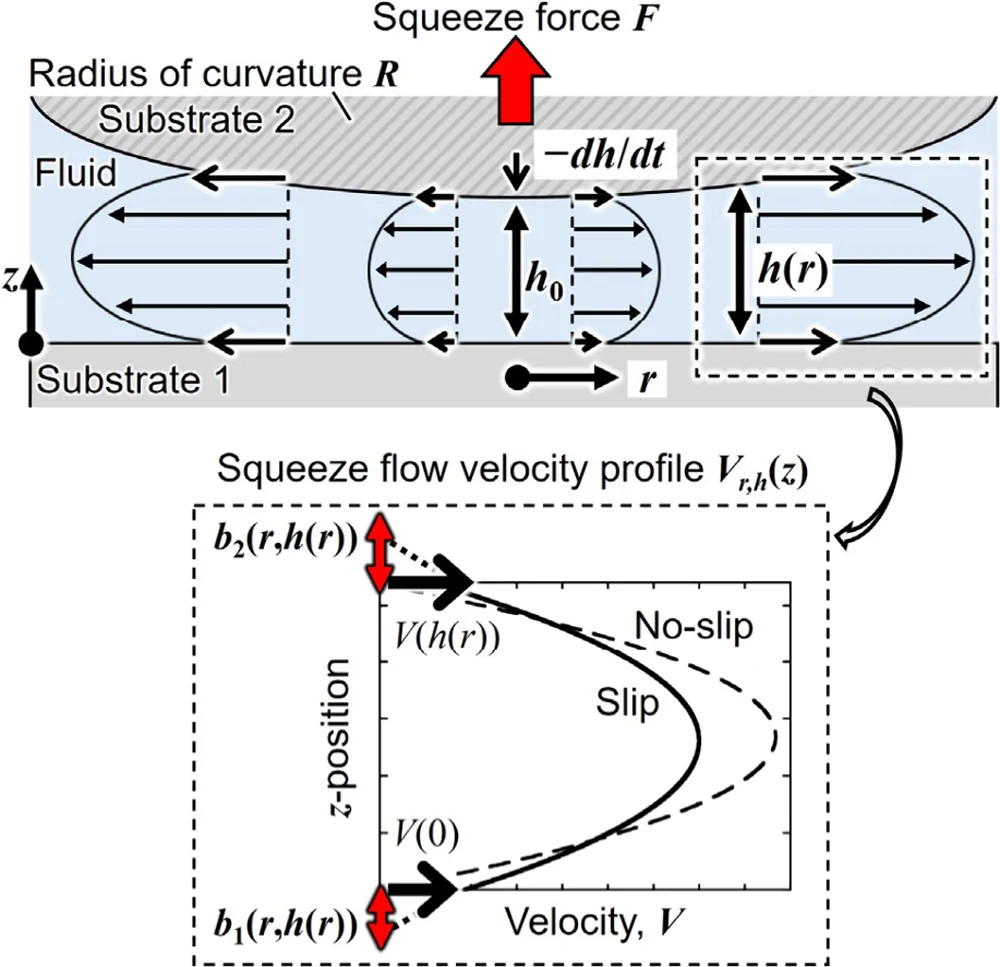
Schematic of the squeeze flow and squeeze force generated within a nanogap. As the gap between the two substrates narrows, the fluid flows radially outward, producing an upward squeeze force. The bottom panel shows the velocity profile at lateral position *r*. The slip lengths at the upper and lower interfaces determine the boundary velocities, which in turn define the velocity profile.

According to the Navier slip boundary condition, the interfacial velocities at the lower and upper substrates, *V*(0) and *V*(*h*(*r*)), respectively, can be expressed as

3 $$V\left(0\right)=b_{1}\left(r,h\left(r\right)\right){\frac{\partial V}{\partial z}|}_{z=0},V\left(h\left(r\right)\right)=-b_{2}\left(r,h\left(r\right)\right){\frac{\partial V}{\partial z}|}_{z=h\left(r\right)}$$

where *b*_1_(*r*, *h*(*r*)) and *b*_2_(*r*, *h*(*r*)) denote the local slip lengths at the lower and upper interfaces, respectively. Assuming axisymmetric flow under the lubrication approximation, we can obtain the velocity profile at lateral position *r* and gap height *h*(*r*), denoted by *V*_*r,h*_(*z*), as follows (Figure 1):^32^

4 $$V_{r,h}\left(z\right)=\left\{\frac{3r}{h{\left(r\right)}^{3}}\left(-\frac{dh}{dt}\right)-\frac{3\left(V\left(0\right)+V\left(h\left(r\right)\right)\right)}{h{\left(r\right)}^{2}}\right\}\left(-z^{2}+h\left(r\right)z\right)+\left(V\left(h\left(r\right)\right)-V\left(0\right)\right)\frac{z}{h\left(r\right)}+V\left(0\right)$$

As illustrated in Figure 1, an increase in the local slip length will increase interfacial velocities *V*(0) and *V*(*h*(*r*)), resulting in a flatter velocity profile.

The gradient of local pressure *p*(*r*) corresponding to this velocity field is given by

5 $$\frac{dp\left(r\right)}{dr}=-\frac{6\eta }{h{\left(r\right)}^{3}}\left\{r\left(-\frac{dh}{dt}\right)-h\left(r\right)\left(V\left(0\right)+V\left(h\left(r\right)\right)\right)\right\}$$

where *η* is the fluid viscosity. Equation 5 indicates that the pressure distribution is affected by local slip lengths *b*_1_(*r*, *h*(*r*)) and *b*_2_(*r*, *h*(*r*)) because interfacial velocities *V*(0) and *V*(*h*(*r*)) are determined by these local slip lengths. Squeeze force *F* is obtained by integrating the pressure distribution over the entire lateral domain:

6 $$F=2\pi \int_{0}^{\infty }p\left(r\right)r\;dr$$

Thus, the local slip lengths affect the overall hydrodynamic response, from the velocity profile and pressure distribution to the macroscopic squeeze force.

In contrast, conventional squeeze-force measurements evaluate slip using a constant slip length *b*_c_, which is assumed to be independent of *r* and *h*(*r*). Under this assumption, the squeeze force is expressed as^23^

7 $$\begin{array}{l} F=G\left(b_{c}\right)\frac{6\pi \eta R^{2}}{h_{0}}\left(-\frac{dh}{dt}\right)\\ G\left(b_{c}\right)=\frac{h_{0}}{3b_{c}}\left[\left(1+\frac{h_{0}}{6b_{c}}\right)\;\ln\left(1+\frac{6b_{c}}{h_{0}}\right)-1\right]\;(b_{c}=\mathrm{constant}) \end{array}$$

where *G*(*b*_c_) is a correction factor accounting for the reduction in hydrodynamic resistance caused by slip. Although constant slip length *b*_c_ obtained from eq 7 represents an effective parameter inferred from the macroscopic force response, it does not directly represent the actual spatially varying slip behavior within the nanogap. In particular, when local shear conditions vary significantly, it remains unclear how the experimentally determined *b*_c_ is related to the underlying distributions of local slip lengths *b*_1_(*r*, *h*(*r*)) and *b*_2_(*r*, *h*(*r*)). Clarifying this relationship is essential for understanding the physical meaning of constant slip length *b*_c_ obtained from conventional squeeze-force measurements.

To this end, we independently quantified local slip lengths *b*_1_(*r*, *h*(*r*)) and *b*_2_(*r*, *h*(*r*)) and constant slip length *b*_c_ within the same experimental system. The local slip lengths were determined from high-resolution measurements of the squeeze-flow velocity using our microscope-based velocimetry technique.^38^ In addition, corresponding squeeze force *F* was calculated via eqs 5 and 6 using the obtained spatial distributions of local slip lengths *b*_1_(*r*, *h*(*r*)) and *b*_2_(*r*, *h*(*r*)) and then compared with the experimentally measured force. Through a quantitative comparison of the flow field and force-based evaluations, the relationship between the spatially distributed local slip lengths and the constant slip length was clarified.

### Experimental Samples and Apparatus

Silicone oil (AR 200, Sigma-Aldrich) was used as the liquid sample. For flow measurements, quantum dots (diameter of 6 nm, fluorescence wavelength of 665 nm) dispersed in toluene were added to the silicone oil at a concentration of 1.0% (v/v). The mixture was stirred at 70 °C for 2 h to ensure a uniform dispersion of the quantum dots in the solvent. The viscosity of the prepared sample (η), measured using a rotational viscometer (TPE-100, Toki Sangyo), was 127 mPa s at 25 °C.

An experimental system was constructed, as shown in Figure 2, to control the gap between a spherical substrate and a flat substrate while measuring both the squeeze flow and the squeeze force. A hemispherical lens sputter-coated with a 100 nm-thick stainless-steel thin film was used as the spherical substrate; the radius of curvature of this lens (*R*) was 15.6 mm. A glass substrate with a thickness of 0.7 mm was used as the flat substrate. The root-mean-square roughness values of the stainless-steel-coated substrate and the glass substrate, measured by atomic-force microscopy (Dimension Icon, Bruker), were 0.9 and 2.2 nm, respectively. The contact angles of the liquid sample on the stainless-steel-coated and glass substrates were 10.3° and 9.1°, respectively.

**Figure 2:**
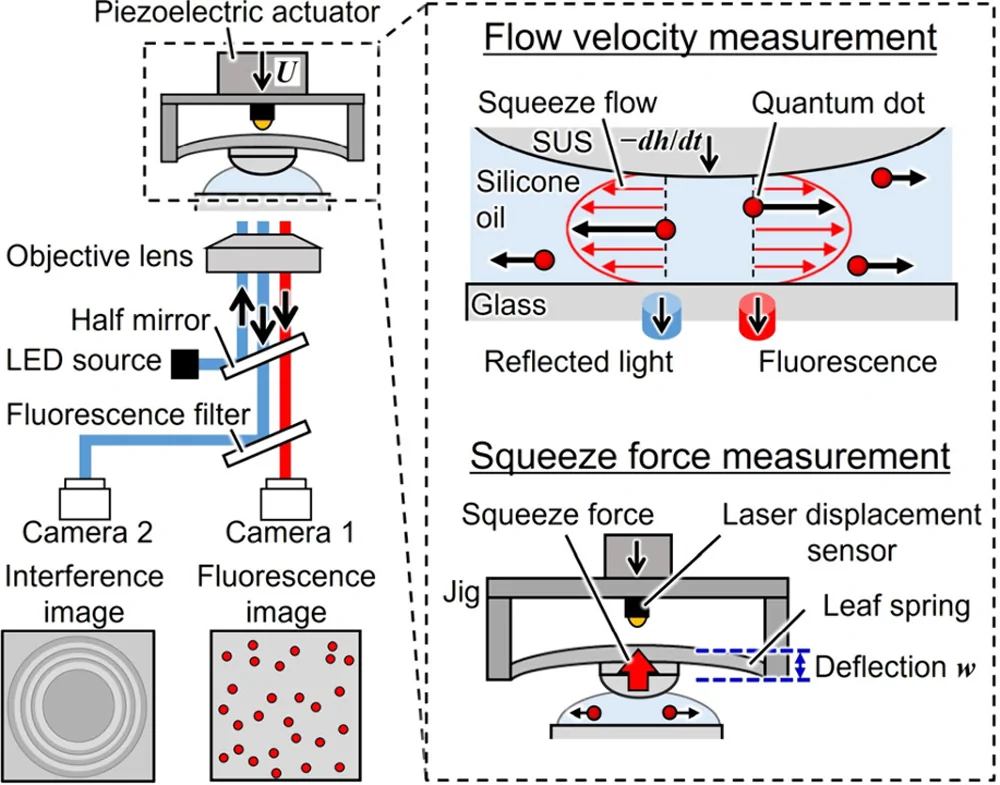
Experimental setup for measuring squeeze-flow velocity and squeeze force. The piezoelectric actuator is driven downward to lower the hemispherical lens through a jig, thereby narrowing the gap between the substrates. The gap is measured from the optical interference image, while the squeeze-flow velocity and squeeze force are measured from the fluorescence image of quantum dots and the deflection of the leaf spring, respectively.

The hemispherical substrate was attached to a leaf spring made of SUS304CSP with a thickness of 0.2 mm. Both ends of the leaf spring were attached to a jig connected directly to a piezoelectric actuator. Deflection *w* of the leaf spring was measured using a spectral interference laser displacement sensor (SI-F01U, KEYENCE). Normal squeeze force *F* was then calculated using the equation *F* = *kw*, where *k* is the spring constant of the leaf spring. Gap height *h*(*r*) and flow fields were observed using an inverted microscope (IX73, Olympus). Excitation light from an LED light source with a wavelength of 460 nm was directed onto the sample through a half-mirror and an objective lens with a magnification of 60× (LUCPLFLN60, Olympus). Gap measurements were performed by capturing optical interference images formed by reflections from the glass and stainless-steel-coated substrates using an sCMOS camera (CS2100M-USB, Thorlabs). Flow measurements were performed by recording the fluorescence images of the quantum dots through a fluorescence filter using an electron-multiplying charge-coupled device (EMCCD) camera (iXon Ultra 897, Andor). The acquisition timing of the laser displacement sensor and the two cameras was synchronized using an external trigger signal.

### Experimental Procedures

A 10 μL droplet of the liquid sample was introduced between the upper and lower substrates. The gap between the substrates was reduced by applying a voltage to the piezoelectric actuator, which moved vertically downward at a constant velocity *U*, generating both squeeze flow and squeeze force.

For flow measurements, actuator velocity *U* was set to 1200 nm/s. Gap distribution *h*(*r*) was obtained by analyzing optical interference images, while radial flow velocity *V*(*r*) was determined by analyzing fluorescence images using particle image velocimetry (PIV). The EM gain of the EMCCD camera was set to 500. The exposure times of the sCMOS and EMCCD cameras were 5 and 25 ms, respectively, and the imaging interval was 25 ms for both of these cameras. Gap and flow velocity analyses were conducted using ImageJ following our previously reported procedure.^38^ In the PIV analysis, the interrogation window size was set to 24 × 24 pixels with an overlap ratio of 50%. Because the pixel size was 0.27 μm/pixel, the resulting lateral resolution was 3.2 μm. Each flow experiment was repeated five times under identical conditions to ensure reproducibility.

For squeeze-force measurements, actuator velocity *U* was set to 600, 800, and 1200 nm/s. The gap distribution was determined by analyzing optical interference images in the same manner as that used for the flow measurements. Deflection *w* of the leaf spring was recorded every 1 ms by using the laser displacement sensor and averaged over intervals of 25 ms. The squeeze force at each gap was calculated from measured *w* and spring constant *k*. Spring constant *k* was calibrated using known applied forces (Supporting Information). Each force measurement was repeated five times under the same conditions.

## Results and Discussion

### Measurement of Gap Height and Narrowing Rate

As expressed in eqs 4 and 7, the velocity profile and squeeze force both depend on gap height *h*(*r*) and gap-narrowing rate −d*h*/d*t*. In our experimental setup, the gap varied continuously owing to the motion of the piezoelectric actuator and the deflection of the leaf spring. Therefore, an accurate determination of *h*(*r*) and −d*h*/d*t* is essential for the quantitative analysis of the flow field and force response.

Figure 3a shows a typical optical interference image obtained during gap narrowing. Figure 3b shows gap distribution *h*(*r*) obtained from the light intensity profile in the region enclosed by the white box in Figure 3a. The light intensity was converted into gap values using an intensity–gap calibration curve obtained following a previously reported procedure.^42^ Gap height *h*(*r*) at each time was determined by fitting eq 1 to the measured gap distribution with *h*_0_ as the fitting parameter. From the root-mean-square error between the gap distribution described by eq 1 and the measured gap distribution, the measurement error of the gap was estimated to be 6 nm.

**Figure 3:**
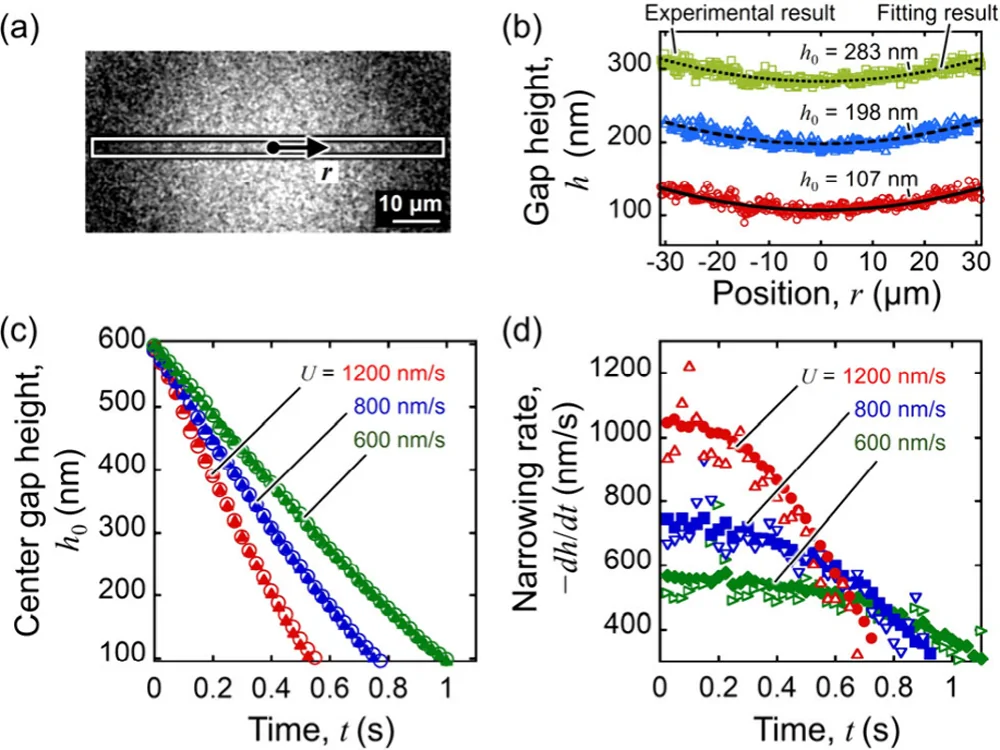
Measurement of the gap height and narrowing rate. (a) Typical optical interference image. (b) Gap distribution *h*(*r*). The symbols represent the gap values extracted from the light intensity of optical interference images, such as that shown in panel a. The lines represent the theoretical gap distributions. (c and d) Time variations in center gap height *h*_0_(*t*) and gap-narrowing rate −d*h*/d*t*, respectively. Empty symbols represent the values obtained by optical interferometry in both panels. Filled symbols indicate the values calculated from deflection *w*(*t*) of the leaf spring.

Figure 3c shows the temporal variation in center gap height *h*_0_(*t*) for actuator velocities *U* of 600, 800, and 1200 nm/s. Under all conditions, the gap decreased monotonically with time. The gap-narrowing rate can be obtained from the temporal variation in the center gap height using

8 $$-\frac{dh}{dt}=-\frac{h_{0}\left(t+\Delta t\right)-h_{0}\left(t-\Delta t\right)}{2\Delta t}$$

However, as shown in Figure 3d, the narrowing rate obtained by optical interferometry exhibited large fluctuations. Because the gap measurement error (6 nm) was comparable to the gap change during a single sampling interval, numerical differentiation amplified the measurement noise. The resulting error in the gap-narrowing rate was estimated to be approximately 250 nm/s by dividing the gap measurement error by time interval Δ*t*.

To improve the measurement accuracy, the gap-narrowing rate was alternatively determined from actuator velocity *U* and deflection *w*(*t*) of the leaf spring. From the geometric relationship among the actuator displacement, spring deflection, and gap height, center gap height *h*_0_ can be expressed as *h*_0_(*t*) = *h*_0_(0) – *Ut* + *w*(*t*). Deflection *w*(*t*) of the leaf spring was measured simultaneously with the optical interference images using a laser displacement sensor. Initial gap *h*_0_(0) was determined by minimizing the root-mean-square error between the calculated *h*_0_(*t*) and *h*_0_(*t*) obtained by optical interferometry. As shown in Figure 3c, *h*_0_(*t*) obtained using this approach is in good agreement with that obtained by optical interferometry. The gap-narrowing rate is then calculated as follows:

9 $$-\frac{dh}{dt}=U-\frac{dw}{dt}=U-\frac{w\left(t+\Delta t\right)-w\left(t-\Delta t\right)}{2\Delta t}$$

This method substantially reduced the fluctuations in the gap-narrowing rate, as shown in Figure 3d. The deflection measurement error of the laser displacement sensor was 0.7 nm, which corresponds to an error of approximately 27 nm/s in the gap-narrowing rate when divided by sampling time interval Δ*t*. Figure 3d further shows that the gap-narrowing rate decreases as the gap narrows. The reduction was more pronounced at higher actuator velocities. This behavior is attributed to the greater hydrodynamic squeeze force generated at higher actuator velocities, which increased the deflection of the leaf spring and consequently reduced the actual gap-narrowing rate.

### Shear-Rate Dependence of Local Slip Lengths

Squeeze-flow velocities were measured using PIV based on the fluorescence images of quantum dots acquired within the narrowing gap between the substrates. Velocity profile *V*_*r,h*_(*z*) was analyzed based on the measured flow velocity distribution at each lateral position *r* and gap height *h*(*r*), and interfacial velocities *V*(0) and *V*(*h*(*r*)) were obtained. Details of the analysis are provided in the Supporting Information. Local slip lengths *b*_1_(*r, h*(*r*)) and *b*_2_(*r, h*(*r*)) were calculated using eq 3. For example, when *h*(*r*) = 127 nm (*h*_0_ = 114 nm; *r* = 20 μm), *b*_1_ and *b*_2_ were 12 and 14 nm, respectively. In the present method, interfacial velocities *V*(0) and *V*(*h*(*r*)) can be determined with a resolution of several micrometers per second (Supporting Information). Therefore, in principle, the slip lengths at the upper and lower substrates can be evaluated independently when their surface properties differ substantially. In the present experiment, however, the difference between the obtained slip lengths *b*_1_ and *b*_2_ was relatively small. This small difference may be attributed to the similar contact angles of the two substrates and to their surface roughness being sufficiently small relative to the gap height. The same analysis was applied to center gap heights ranging from 99 to 293 nm and lateral positions ranging from 15 to 35 μm. The measurement error in the slip length obtained using this method was estimated to be 4 nm (Supporting Information).

Figure 4a shows the relationship between the local slip lengths and gap height *h*(*r*) at *r* = 30 μm. At both interfaces, slip occurred when the gap decreased below 260 nm. Although the slip lengths differed between the upper and lower interfaces, they increased as the gap narrowed and eventually approached constant values. Figure 4b shows the relationship between the local slip length and lateral position *r* when *h*_0_ = 114 nm. At both interfaces, the slip lengths increased with *r*. These results indicate that the slip length is not a constant quantity but depends on the lateral position and gap height. To elucidate this spatial variation, apparent local shear rate *γ̇* at the interface was calculated using eq 2, and its relationship with the local slip length was evaluated.

**Figure 4:**
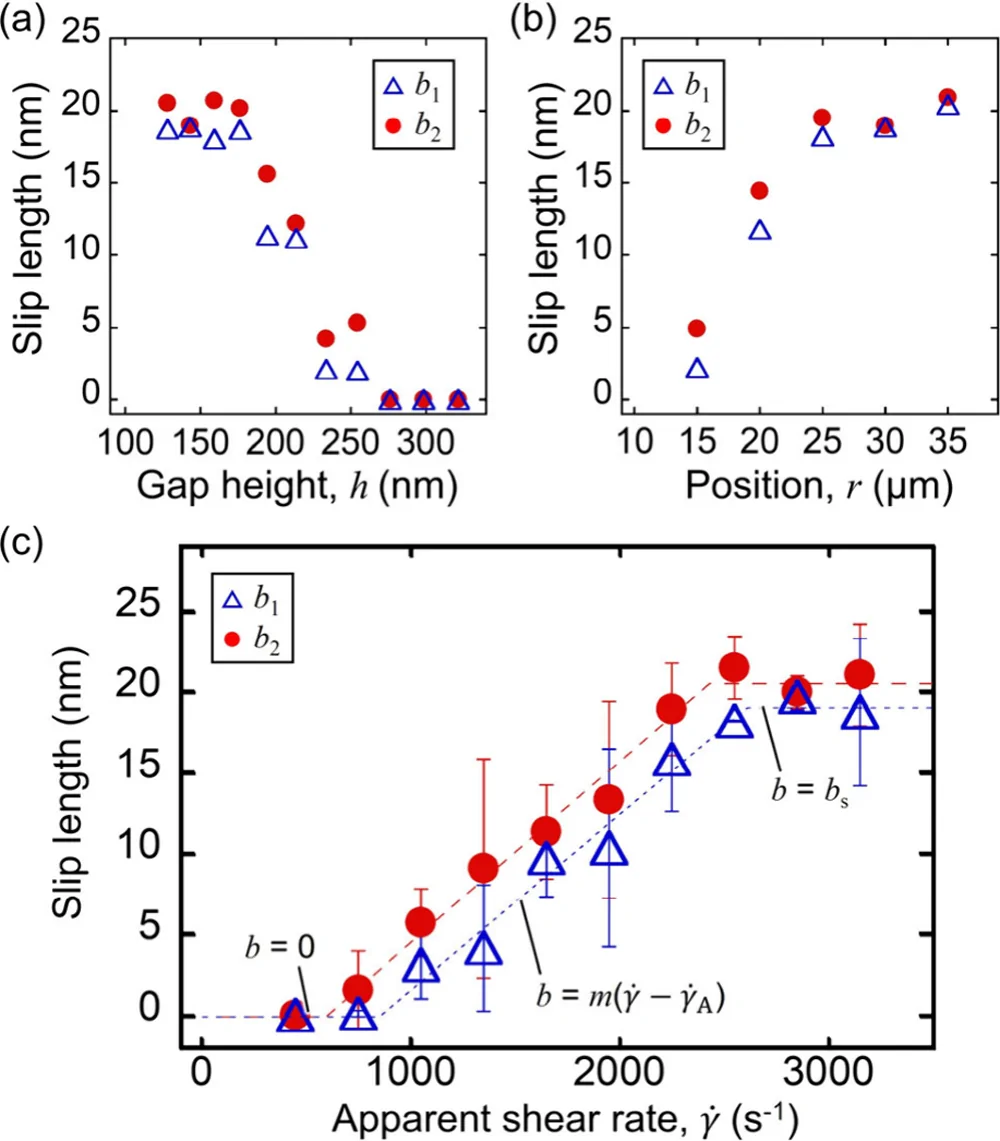
Measurement results of the local slip lengths. (a) Relationship between slip length and gap height *h*(*r*) at *r* = 30 μm. (b) Relationship between slip length and lateral position *r* for *h*_0_ = 114 nm. (c) Relationship between slip length and apparent shear rate *γ̇* at the interface. Error bars represent the standard deviation. The dotted lines represent the approximate results for the low-shear-rate, transition, and high-shear-rate regimes.

As shown in Figure 4c, the local slip lengths obtained at different gap heights *h*(*r*) and lateral positions *r* collapsed onto a single master curve when plotted against the local shear rate at the interface, exhibiting three distinct regimes. At low shear rates, no slip occurred, and the slip lengths were zero (*b*(*r*, *h*(*r*)) = 0). When the shear rate exceeded critical values, slip occurred, and the slip lengths increased linearly with shear rate *b*(*r, h*(*r*)) = *m*(*γ̇* – *γ̇*_A_), where *m* and *γ̇*_A_ are fitting parameters. At higher shear rates, the slip lengths saturated at constant values (*b*(*r*, *h*(*r*)) = *b*_s_). The saturated slip lengths were 19 and 21 nm for the lower and upper interfaces, respectively. The relationships between the slip length and shear rate in these regimes are empirical approximations introduced to describe the trends observed in the experimental results. In addition, no clear gap height dependence of the relationship shown in Figure 4c was observed (Supporting Information).

The existence of a critical shear rate at which slip occurs has been discussed in previous studies.^32,43,44^ For example, Spikes and Granick^39^ proposed a model in which slip occurs when the shear rate exceeds a critical value, and the slip length remains constant thereafter. In contrast, our results reveal a transition regime (*γ̇* ∼ 600–2600 s^–1^) between slip onset and saturation, where the slip length increases linearly with shear rate.

The local slip length obtained in this study is a continuum-mechanics parameter defined from the Navier boundary condition expressed in eq 3. Previous studies have discussed the applicability of continuum fluid-mechanics models to flows in nanogaps.^12^ The gap heights investigated in this study, ranging from 100 to 300 nm, are much larger than the molecular-length scale of the silicone oil. Therefore, under the present experimental conditions, it is reasonable to describe the flow within the gap using a continuum fluid-mechanics model and to apply the Navier slip boundary condition at the solid–liquid interfaces. In terms of interfacial friction coefficient *κ*, which is defined as the ratio of the shear stress at the interface to the interfacial velocity, the slip length can be expressed as *b* = *η*/*κ*.^11^ The squeeze force was measured over a gap height range of 100–300 nm at a sufficiently low actuator velocity, for which interfacial slip was negligible. The measured force agreed well with the theoretical prediction assuming a constant viscosity, indicating no clear gap height dependence of the effective viscosity over this range (Supporting Information). Therefore, the increase in the slip length within the transition regime suggests that interfacial friction coefficient *κ* decreased with an increase in shear rate. This decrease in *κ* may be attributed to shear-induced changes in the liquid structure, including a decrease in the local fluid density near the wall,^45,46^ degradation in the interfacial layer structure and molecular orientation,^47,48^ and shortening of the correlation time of intermolecular interactions between solid and liquid molecules.^11,49^ Such changes may reduce the tangential momentum transfer across the solid–liquid interface, thereby decreasing *κ*. The saturation of the slip length at high shear rates suggests that the interfacial structure reached a steady state and that *κ* converged to a constant value.

Similar behavior has been reported in previous molecular dynamics simulation studies.^50^ For example, Shuvo et al.^51^ and Martini et al.^40^ reported, using water and *n*-decane, respectively, that the slip length increases with shear rate and approaches a constant value at high shear rates. The observation of similar trends in the present study and previous studies suggests that the three-regime description of the shear-rate dependence of slip length may provide a useful framework applicable to different material systems. However, the parameters characterizing this shear-rate dependence are expected to depend not only on the interactions between the solid and liquid materials but also on surface properties and experimental conditions. For example, the critical shear rate for the onset of slip has been reported to depend on surface roughness^26^ and temperature.^52^ Therefore, critical shear rate *γ̇*_A_, slope in the transition regime *m*, and saturation slip length *b*_s_ are not universal constants but should instead be treated as system-specific parameters that depend on the combination of solid and liquid materials, surface properties, and experimental conditions. In addition, as shown in Figure 4c, the slip lengths obtained in this study exhibited relatively large scatter in the transition regime. This scatter may have arisen from local variations in surface properties and interfacial conditions. In the transition regime, where the interfacial state changes from the no-slip state toward the saturated-slip state, local differences in surface and interfacial conditions may be more readily reflected in the slip length. Therefore, such local heterogeneities may have contributed to the observed scatter in the slip length.

### Spatial Distribution of Local Slip Length and Its Influence on Squeeze Force

The shear-rate-dependent slip-length relationship shown in Figure 4c was used to predict the spatial distribution of the local slip lengths and its influence on the pressure and force responses. Figure 5a shows the lateral distribution of apparent shear rate *γ̇* at the interface for each *h*_0_, calculated using eq 2. The apparent shear rate varied significantly depending on both center gap height *h*_0_ and lateral position *r*. Because the shear rate is proportional to *r*/*h*(*r*)^2^, the shear rate initially increased with *r*, whereas the simultaneous increase in gap height *h*(*r*) suppressed the shear rate at a higher *r*. Consequently, it peaked at a certain lateral position *r*. Using the local shear-rate distributions shown in Figure 5a and the experimentally obtained shear-rate dependence of the local slip length shown in Figure 4c, we calculated the lateral distributions of the local slip lengths for each *h*_0_, as shown in panels b and c of Figure 5. The slip length at each interface was approximated as *b*(*r*, *h*(*r*)) = 0 in the low-shear regime, *b*(*r*, *h*(*r*)) = *m*(*γ̇* – *γ̇*_A_) in the transition regime, and *b*(*r*, *h*(*r*)) = *b*_s_ in the high-shear regime.

**Figure 5:**
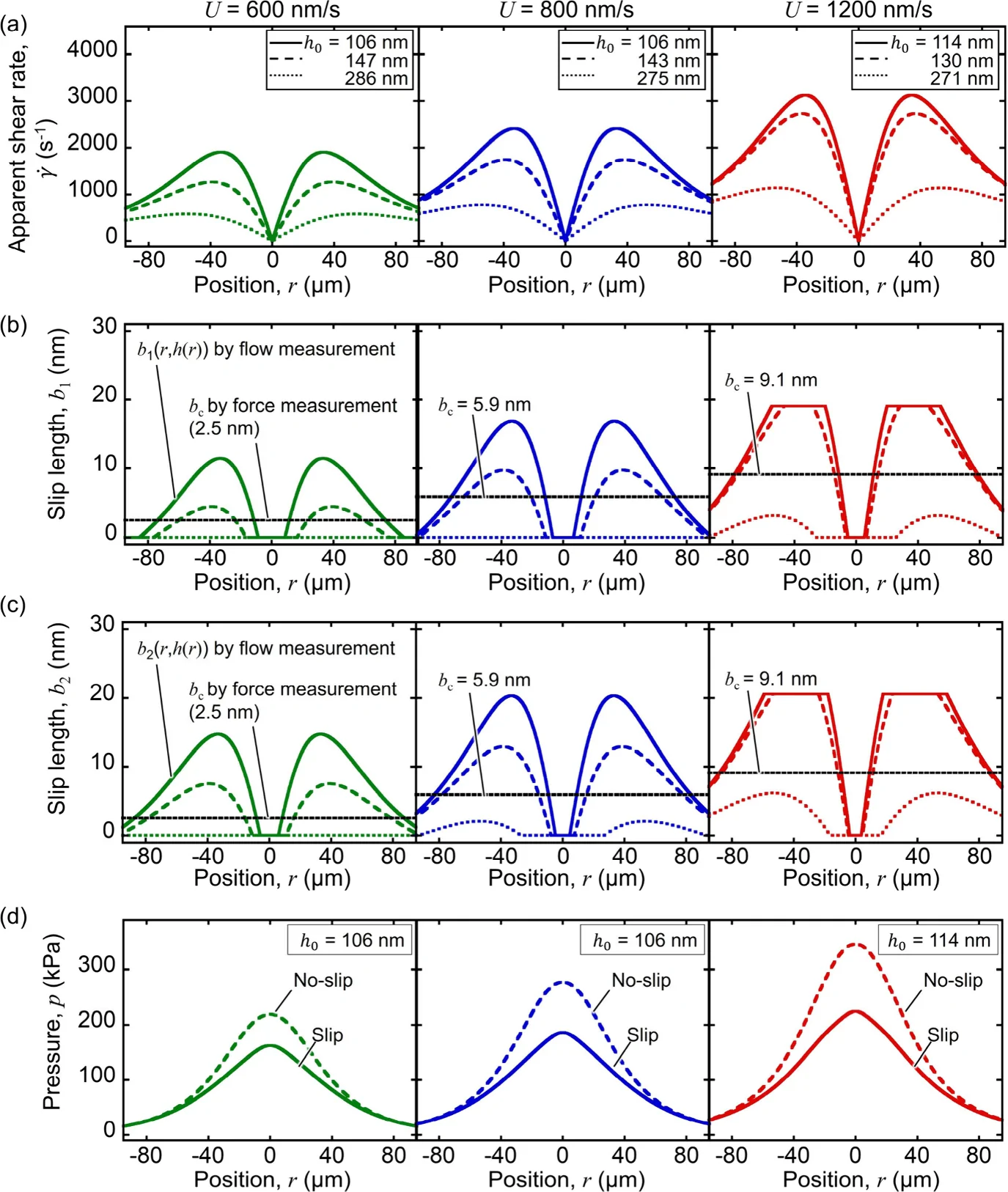
Spatial distribution for various actuator velocities *U* of (a) apparent shear rate *γ̇* at the interface, (b and c) local slip lengths *b*_1_ and *b*_2_, obtained by considering the shear-rate dependence of the slip length shown in Figure 4c, and (d) pressure *p*(*r*). For each *U*, panels a–c show the results obtained at the same center gap heights *h*_0_. With a decrease in *h*_0_ and an increase in *U*, the shear rate increases, leading to large slip lengths that in some regions reach saturation. Constant slip length *b*_c_ in panels b and c for each *U* represents the value obtained from the squeeze force (Figure 6) using the conventional constant-slip-length model.

As shown in panels b and c of Figure 5, at low actuator velocity *U* and large center gap height *h*_0_, no slip occurs because the shear rate remains below the critical value throughout the lateral region. With an increase in *U* and a decrease in *h*_0_, the shear rate exceeded the critical value in certain lateral regions, resulting in the emergence of finite slip lengths. At even higher *U* and lower *h*_0_ values, the high-shear-rate regime expanded and the area where the slip length reached its saturated value increased. These results demonstrate that the local slip length is not constant but exhibits a pronounced spatial distribution governed by local shear conditions.

The obtained spatial distributions of the local slip length were substituted into eqs 3 and 5 to calculate pressure gradient d*p*(*r*)/d*r*. Pressure distribution *p*(*r*) was then obtained by numerically integrating d*p*(*r*)/d*r* along the *r* direction. The integration range was set to −*R*/5 ≤ *r* ≤ *R*/5 (*R*/5 = 3.1 mm), and the boundary condition *p* = 0 was imposed at *r* = ±*R*/5. Figure 5d shows the resulting pressure distributions, where the dashed line indicates the pressure distribution calculated under the no-slip condition. When local slip was incorporated, the pressure gradient was reduced in the lateral regions where slip occurred according to eq 5, leading to a substantial decrease in pressure throughout the high-pressure region. Figure 6 shows the squeeze force calculated by integrating the pressure distribution using eq 6. The results show that, at higher *U* and lower *h*_0_ values, the reduction in pressure due to slip became more pronounced, leading to a substantial decrease in the squeeze force compared with that under the no-slip condition.

**Figure 6:**
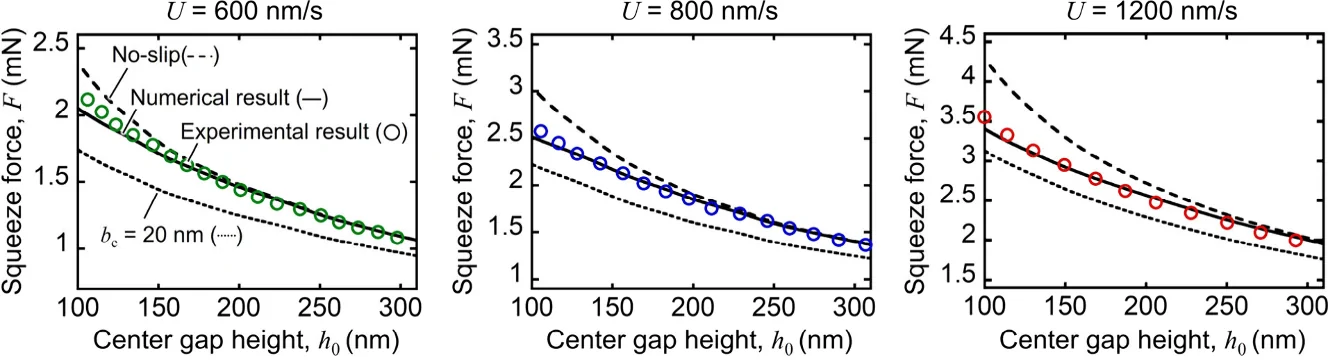
Relationship between the squeeze force and center gap height *h*_0_. Symbols represent the experimental results. The solid lines indicate the numerical results based on the shear-rate dependence of the slip length. The dashed and dotted lines indicate the theoretical results calculated from eq 7 under the no-slip condition (*G* = 1) and using the saturated slip length as the constant slip length (*b*_c_ = 20 nm), respectively.

To validate the numerical analysis, the calculated squeeze forces were compared with the experimental results. The circular symbols in Figure 6 represent the measured squeeze forces. The calculated results were in good agreement with the experimental values for all actuator velocities. The root-mean-square error between the calculated and experimental results in the *h*_0_ range of 100–300 nm was approximately 0.05 mN for all *U* values. For comparison, the dotted line in Figure 6 shows the prediction made by the conventional constant-slip-length model (eq 7), in which the average saturated slip length at the two interfaces (*b*_s_ = 20 nm) was used as constant slip length *b*_c_. This model failed to reproduce the experimentally measured squeeze force. These results indicate that the shear-rate dependence of the local slip length in the transition regime plays a significant role in determining the force response and cannot be neglected. As a result, the macroscopic squeeze force can be quantitatively predicted from the spatial distribution of the local slip length determined by the local shear condition. Therefore, the experimentally observed force response is a direct consequence of the spatially distributed slip behavior within the nanogap. Furthermore, the successful reproduction of the measured squeeze force confirms the validity of using the local-slip-length distributions obtained from the flow measurements to predict hydrodynamic force responses at the macroscopic level.

### Comparison between Local and Constant Slip Lengths

In conventional methods based on squeeze-force measurements, the slip length is evaluated as a spatially uniform quantity *b*_c_ using eq 7. In contrast, this study demonstrated that local slip length *b*(*r*, *h*(*r*)) varies significantly with lateral position *r* and gap height *h*(*r*). Therefore, we examined the relationship between constant slip length *b*_c_ obtained from the force measurements and spatially distributed local slip length *b*(*r*, *h*(*r*)) obtained from the flow measurements.

Figure 7 shows constant slip length *b*_c_ calculated by fitting eq 7 to the force measurement results shown in Figure 6. For each actuator velocity *U*, the fitting was performed over a center gap height *h*_0_ range of 100–300 nm (Supporting Information). The resulting constant slip length *b*_c_ increased with *U*. To compare *b*_c_ with the local slip-length distributions shown in panels b and c of Figure 5, arithmetic mean slip length *b*_ave_ was calculated from the spatial distributions as follows:

10 $$b_{\mathrm{ave}}=\frac{1}{N_{h_{0}}N_{r}}\overset{N_{h_{0}}}{\underset{i=1}{\sum }}\overset{N_{r}}{\underset{j=1}{\sum }}b\left(r_{j},h{\left(r\right)}_{ij}\right)$$

where *N*_*h*_0__ is the number of measurement points for center gap height *h*_0_ within the 100 nm ≤ *h*_0_ ≤ 300 nm range and *N*_*r*_ is the number of measurement points for lateral position *r* within the −*R*/5 ≤ *r* ≤ *R*/5 range, corresponding to the range used in the squeeze-force calculations. As shown in Figure 7, *b*_ave_ is significantly lower than constant slip length *b*_c_. This discrepancy arises because slip occurred only in limited regions where the local shear rate at the interface exceeded the critical value, whereas no slip occurred over a large region. Consequently, arithmetic mean *b*_ave_ was dominated by the extensive no-slip region and therefore underestimated the contribution of the localized slip regions.

**Figure 7:**
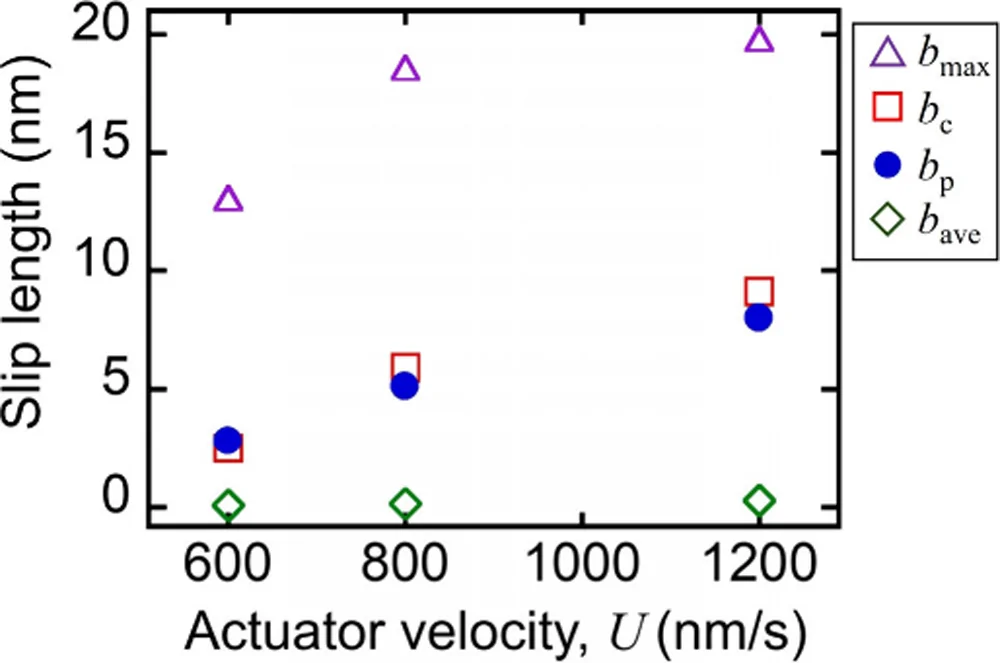
Comparison between constant slip length *b*_c_ and the representative values of the local slip length. Square symbols represent *b*_c_ values, whereas the triangular, diamond, and circular symbols represent maximum *b*_max_, arithmetic mean *b*_ave_, and pressure-weighted average *b*_p_ of the local slip lengths, respectively.

To account for the contribution of each regime to the force response, pressure-weighted average slip length *b*_p_ was calculated as follows:

11 $$b_{p}=\frac{1}{\overset{N_{h_{0}}}{\underset{i=1}{\sum }}\overset{N_{r}}{\underset{j=1}{\sum }}p\left(r_{j},h{\left(r\right)}_{ij}\right)}\overset{N_{h_{0}}}{\underset{i=1}{\sum }}\overset{N_{r}}{\underset{j=1}{\sum }}p\left(r_{j},h{\left(r\right)}_{ij}\right)\times b\left(r_{j},h{\left(r\right)}_{ij}\right)$$

where local pressure *p*(*r*, *h*) serves as the weighting function. As shown in Figure 5d, pressure distribution *p*(*r*, *h*) used in this analysis was calculated from eqs 3 and 5 using the experimentally obtained *b*(*r*, *h*). As shown in Figure 7, the pressure-weighted average slip length *b*_p_ is quantitatively consistent with constant slip length *b*_c_ for all actuator velocities *U*. The relative deviations of *b*_p_ from *b*_c_ were 12%, 14%, and 12% at *U* values of 600, 800, and 1200 nm/s, respectively. Here, *b*_p_ is the average of local slip-length distribution *b*(*r*, *h*) weighted by the corresponding pressure distribution. In contrast, *b*_c_ is an effective slip length determined such that squeeze force *F*(*b*_c_), calculated using a model that assumes a spatially uniform slip length, agrees with the experimentally measured squeeze force. Therefore, eq 11 is not an exact mathematical transformation relating *b*(*r*, *h*) to *b*_c_. Nevertheless, the quantitative agreement between *b*_p_ and *b*_c_ indicates that the constant slip length obtained from squeeze-force measurements is neither simple spatial average *b*_ave_ nor maximum *b*_max_ of the local slip lengths, where *b*_max_ represents the maximum value of the local slip lengths for each *U*. Instead, *b*_c_ is an effective quantity that primarily reflects the local slip state in regions where *p*(*r*, *h*) is high. This interpretation is consistent with eq 6, because the squeeze force is obtained by spatially integrating the local pressure distribution, such that local slip in high-pressure regions contributes more strongly to the macroscopic force response.

These findings clarify the applicability and limitations of slip evaluations based on a constant slip length. Because the constant slip length reflects the integrated influence of local slip on the measured force response, it remains effective for evaluating the relative changes in the interfacial slip behavior and provides a useful macroscopic index for qualitatively comparing the overall degree of slip among different surface materials and coatings.^53,54^ However, caution must be exercised when quantitatively interpreting local slip behavior. When the local slip state varies substantially because of spatial variations in the shear rate at the interface, gap height, viscosity, or surface structure, the constant slip length alone cannot accurately represent the actual local slip distributions at the interface. Different local slip distributions may produce the same constant slip length if their pressure-weighted contributions to the force response are comparable. For example, a highly localized region exhibiting a large slip length and a broader region exhibiting a moderate slip length may generate a similar force response when their pressure-weighted effects are equivalent. Conversely, even for identical surfaces, the constant slip length obtained from the force measurements may differ, depending on the gap height distribution.

In addition to these effects arising from spatially varying local slip, some studies have also discussed factors that may affect the interpretation of slip lengths obtained from conventional force-based measurements, including the choice of hydrodynamic modeling and the influence of surface nanostructures and nanobubbles.^55,56^ Regarding the effects of surface morphology and nanobubbles, the root-mean-square roughness values of the two surfaces used in the present study (0.9 and 2.2 nm) were much smaller than the minimum gap height (100 nm), suggesting that the effect of surface roughness on the local gap height and pressure distributions was limited under the present experimental conditions. Furthermore, because both surfaces exhibited high wettability toward the liquid sample, nanobubbles were unlikely to have a dominant influence. However, when surface roughness or surface structures produce non-negligible variations in the local gap height, the actual gap height distribution should be taken into account when determining *b*(*r*, *h*) and *p*(*r*, *h*) to appropriately calculate *b*_p_ and quantitatively compare it with *b*_c_. Similarly, when nanobubbles are present, their influence on the local flow boundary conditions and the force response should be taken into account. Whether the relationship between *b*_p_ and *b*_c_ observed in the present study remains valid when such effects are non-negligible requires further investigation.

The interpretation presented in this study, in which the constant slip length is regarded as a pressure-weighted average of the local slip lengths, is based on the fact that the measured squeeze force in the squeeze flow is determined by the spatial integration of the local pressure distribution. In contrast, in other nanogap flows, including shear-driven Couette flow and pressure-driven Poiseuille flow, a different weighting function may be required to relate the local slip length to the slip length inferred from macroscopic responses, such as shear force or flow rate. Even in such flow configurations, when the local slip state varies spatially, the slip length inferred from the macroscopic response should be distinguished from the local slip length. Therefore, quantifying the spatial distribution of the local slip length is essential for understanding the physical mechanisms governing interfacial slip and for establishing appropriate boundary conditions for flow fields with spatially varying slip conditions. This is particularly important for nanoscale flows and lubrication systems, where the gap height and shear rate vary substantially with the lateral position.

## Conclusions

We investigated the relationship between the spatial distribution of local slip lengths and the constant slip length by independently measuring the squeeze-flow velocity and the corresponding squeeze force in nanogaps by using the same experimental system. The results revealed that the local slip length varied spatially with the gap height and lateral position and that this variation was governed by the local shear rate at the interface. The local slip length exhibited three distinct regimes with increasing shear rate: a no-slip regime, a transition regime characterized by a linear increase in the slip length, and a high-shear-rate regime in which the slip length saturated at a constant value. The squeeze force calculated on the basis of the relationship between the local slip length and the shear rate was in good agreement with the experimentally measured squeeze force. This result demonstrates that the macroscopic force response in nanogaps is determined by the spatial distribution of local slip lengths. A comparison between the constant slip length obtained from the force measurements and the spatially distributed local slip lengths revealed that the constant slip length is neither the simple average nor the maximum of the local slip lengths but rather a pressure-weighted effective quantity.

Overall, this study clarifies the physical meaning of the constant slip length obtained through conventional squeeze-force measurements and experimentally demonstrates the correspondence between local interfacial slip behavior and the macroscopic force responses in nanoscale flows. These findings provide deeper insights into interfacial boundary conditions in such flows and offer a framework for interpreting slip-length measurements.

## Supplementary Material



## Notes

Substrate surface topography measurements, piezoelectric actuator velocity, spring constant determination for force measurements, evaluation of the spring deflection distribution, detailed slip-length measurement procedure, slip-length dependence on local shear rate at different gap heights, constant-slip-length determination, gap height dependence of effective viscosity, and squeeze-force measurement using a sample without quantum dots (PDF)

## Abbreviations

PIV: particle image velocimetry
sCMOS: scientific complementary metal oxide semiconductor
EMCCD: electron-multiplying charge-coupled device
