# Supplementary material for: Linking Flow-Derived Local Slip Lengths to Force-Derived Effective Slip Lengths in Nanogaps
Source: Langmuir. 2026 Oct 26;42(44):32468–77. doi: 10.1021/acs.langmuir.6c03687 (PMC13757955; doi:10.1021/acs.langmuir.6c03687)
Supplement: Supplementary file 1 [file la6c03687_si_001.pdf]

Supporting Information

Linking Flow-Derived Local Slip Lengths to Force-Derived Effective Slip Lengths in Nanogaps

Hidetaka Ozeki [1*], *Naoki Azuma* [1*], *Kenji Fukuzawa* [1], *Kento Shimizu* [1], *Yuxi Song* [2], *Shintaro Itoh* [1], *Hedong Zhang* [2]

[1] Department of Micro-Nano Mechanical Science and Engineering, Nagoya University, Furo-cho, Chikusa-ku, Nagoya, 464-8603, Japan

[2] Department of Complex Systems Science, Nagoya University, Furo-cho, Chikusa-ku, Nagoya, 464-8601, Japan

E-mail: *(N.A.) naoki.azuma@mae.nagoya-u.ac.jp
*(H.O.) ozeki.hidetaka.n4@s.mail.nagoya-u.ac.jp

Number of pages: 17
Number of figures: 11

Contents:

1. Evaluation of Substrate Surface Roughness by Atomic Force Microscopy S2
2. Piezoelectric Actuator Velocity S2
3. Calibration of the Leaf-Spring Constant S3
4. Evaluation of the Deflection Distribution of the Leaf Spring in Squeeze-Force Measurements S5
5. Quantification of the Local Slip Length S7
6. Relationship between Slip Length and Local Shear Rate at Different Gap Heights S13
7. Obtaining Constant Slip Length from Squeeze-Force Measurements S14
8. Gap-Height Dependence of Effective Viscosity S15
9. Squeeze-Force Measurement Using a Sample without Quantum Dots S16
REFERENCE S17

1. Evaluation of Substrate Surface Roughness by Atomic Force Microscopy

Figure S1 shows the surface topographies of the upper and lower substrates obtained by atomic force microscopy. No distinct surface structures were observed on either surface. The root-mean-square roughness values of the SUS-coated and glass surfaces were 0.9 and 2.2 nm, respectively.

2. Piezoelectric Actuator Velocity

To confirm that the piezoelectric actuator operated at a constant speed, the signal from the capacitance sensor integrated into the actuator was recorded. The actuator was operated under closed-loop control. Figure S2 shows the actuator displacement calculated from the capacitance-sensor output when the piezoelectric actuator was driven at U = 1200 nm/s according to the experimental procedure. Linear fitting yielded a velocity of 1199.7 ± 0.1 nm/s, corresponding to a difference of 0.3 nm/s from the set value. The velocity variation was also 0.1 nm/s. Both values were sufficiently smaller than the measurement error of the gap-narrowing rate in this study. Therefore, under the present experimental conditions, the actuator was driven at a constant speed consistent with the set value, and the influence of actuator-velocity variations on the analysis results was negligible.

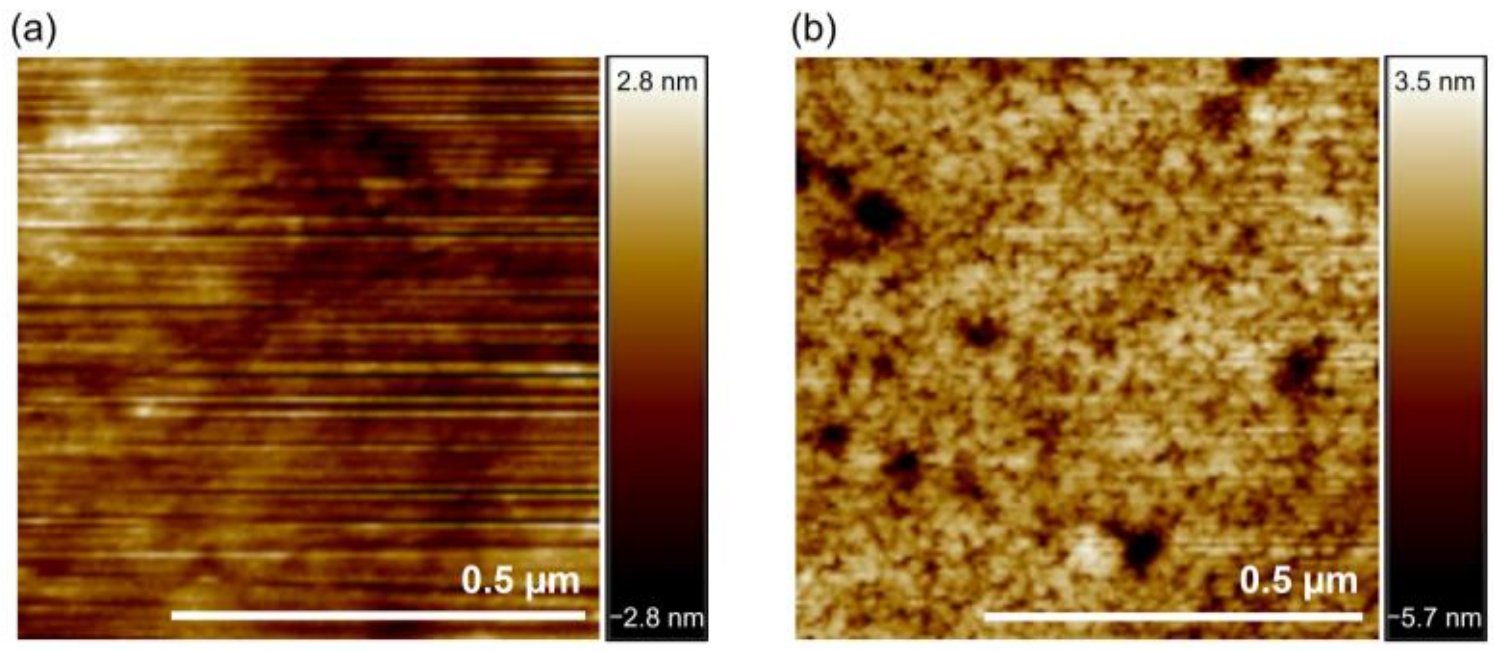

Figure S1. Surface topographies obtained by atomic force microscopy: (a) SUS-coated hemispherical lens and (b) glass substrate.

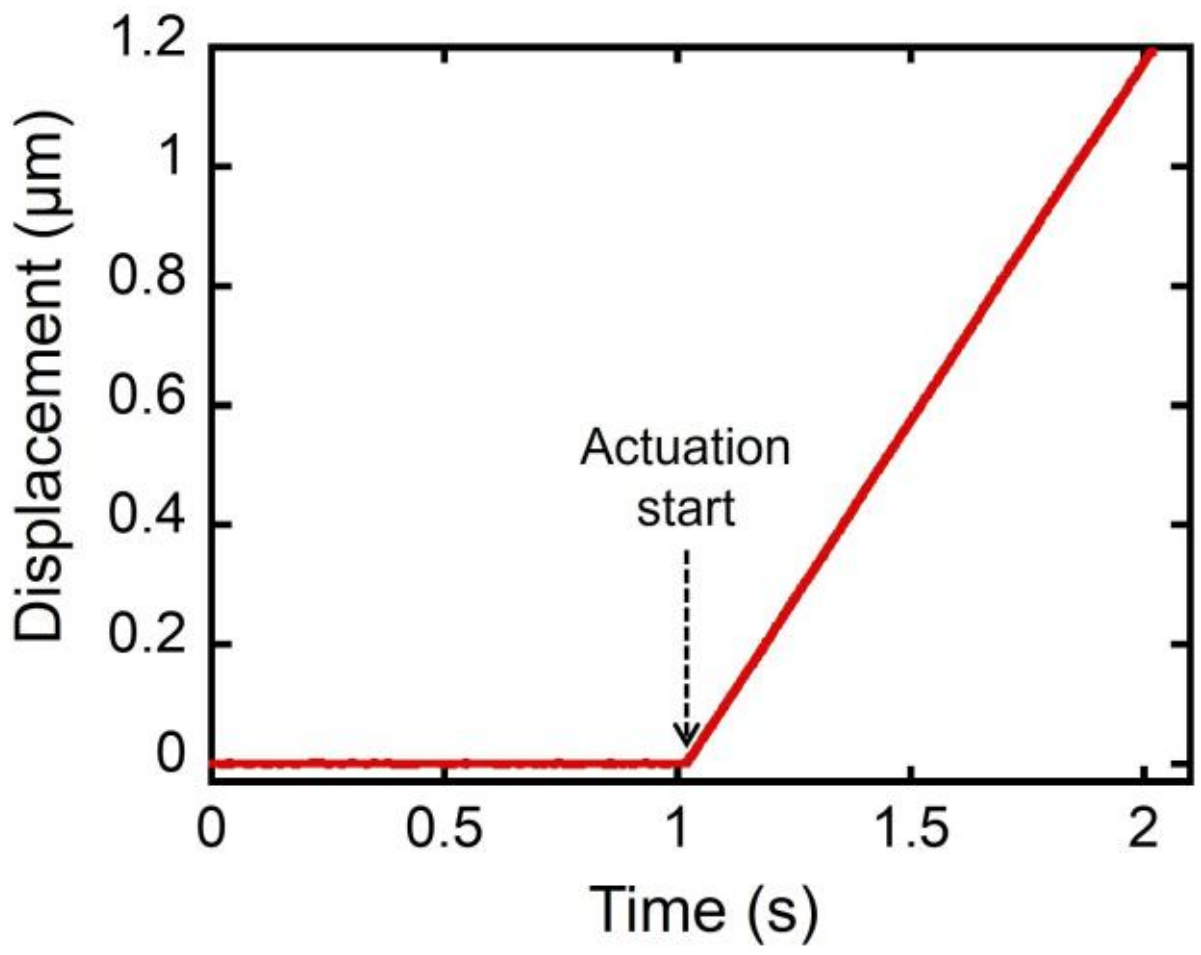

Figure S2. Time variation of the actuator position at $U = 1200$ nm/s. The arrow indicates the start of actuator motion and the subsequent data were used for linear fitting.

3. Calibration of the Leaf-Spring Constant

The spring constant k of the leaf spring used to measure the squeeze force was determined based on a method proposed in a previous study.[S1] Under the no-slip condition,

the squeeze force F at each center gap height h_0 is given by the following equation, which corresponds to Eq. (7) with $G(b_c) = 1$:

$$F(h_0) = \frac{6\pi\eta R^2}{h_0}\left(-\frac{dh}{dt}\right) \tag{S1}$$

When the leaf spring is deflected by a deflection w due to the squeeze force, force equilibrium gives:

$$-\frac{w}{dh/dt} = \frac{6\pi\eta R^2}{k}\frac{1}{h_0} \tag{S2}$$

From Eq. (S2), $-w/(dh/dt)$ is proportional to $1/h_0$ under the no-slip condition. Therefore, the relationship between $-w/(dh/dt)$ and $1/h_0$ was obtained, and the spring constant k was calculated from the slope in the no-slip region. Figure S3 shows the relationship between $-w/(dh/dt)$ and $1/h_0$ for each actuator velocity U of the piezoelectric actuator, where the leaf-spring deflection w was measured using the laser displacement sensor. For each actuator velocity U, linear fitting was performed over a range of center gap heights h_0 where the local shear rate at the interface throughout the lateral region was below the critical shear rate for slip onset shown in Fig. 4(c). The spring constant k obtained for actuator velocities of 600, 800, and 1200 nm/s was 16.8 ± 0.1 kN/m, indicating that k was nearly independent of the actuator velocity.

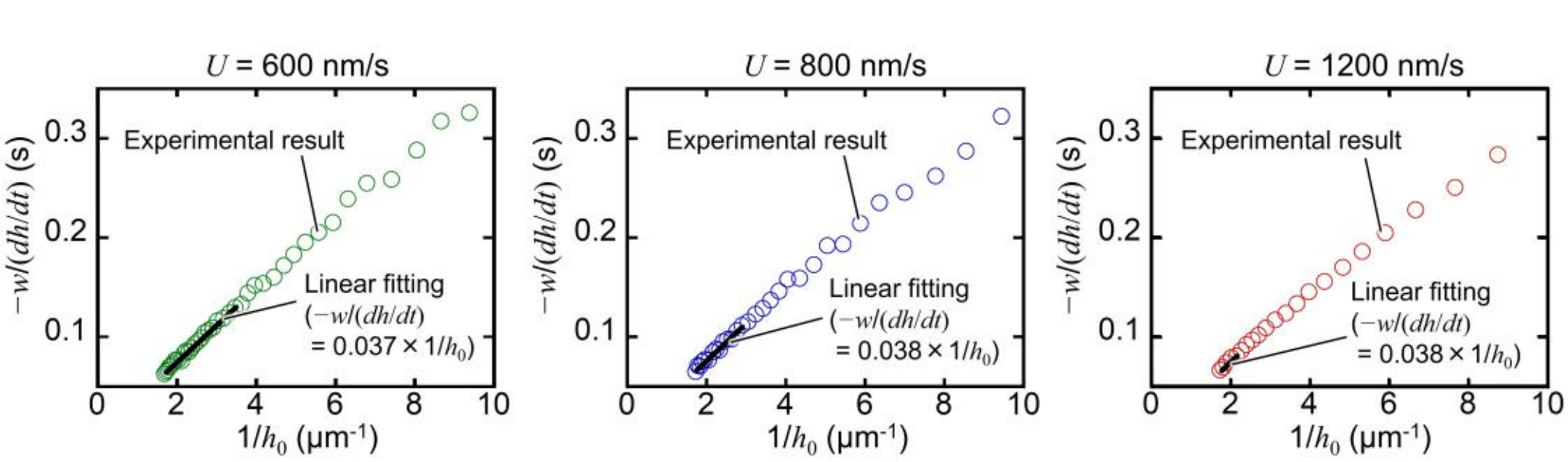

Figure S3. Relationship between $-w/(dh/dt)$ and $1/h_0$, where w is the leaf-spring deflection, and $(-dh/dt)$ is the gap-narrowing rate. The solid lines represent linear fits over

the region where the slip could be assumed to be absent. The spring constant is calculated from the slope obtained by fitting.

4. Evaluation of the Deflection Distribution of the Leaf Spring in Squeeze-Force Measurements

Figure S4 shows the squeeze-force measurement system. Both ends of the leaf spring were fixed to the jig, and the laser displacement sensor was positioned to measure the leaf-spring deflection at the center of the hemispherical lens, which was mounted at the midpoint of the leaf spring. The red dot in Fig. S4 indicates the position measured by the displacement sensor. In this system, the lateral deflection distribution of the leaf spring $W(x)$ is expressed as follows:

$$W(x) = w\left\{16\left|\frac{x}{l}\right|^3 - 12\left(\frac{x}{l}\right)^2 + 1\right\} \quad \left(-\frac{l}{2} \leq x \leq \frac{l}{2}\right) \tag{S3}$$

Here, as shown in Fig. S4, the center of the leaf spring is defined as $x = 0$, and w and l denote the maximum deflection ($W(x = 0)$) and the total length of the leaf spring, respectively. In the squeeze-force measurements performed in this study, the maximum value of w over the measurement range was approximately 200 nm. Under this condition, Eq. (S3) gives a difference of 0.8 nm between the deflection at $x = \pm$ 0.5 mm and that at the center ($x = 0$). As shown in Fig. S4, the measurement position was located near the center of the hemispherical lens. Even if the measurement position were displaced by 0.5 mm from the center, the maximum measurement error arising from the deflection distribution of the leaf spring would be 0.8 nm. Because this value is comparable to the measurement error of the laser displacement sensor (0.7 nm), the effects of the leaf-spring

deflection distribution and positional misalignment on the squeeze-force measurements were considered sufficiently small under the present experimental conditions.

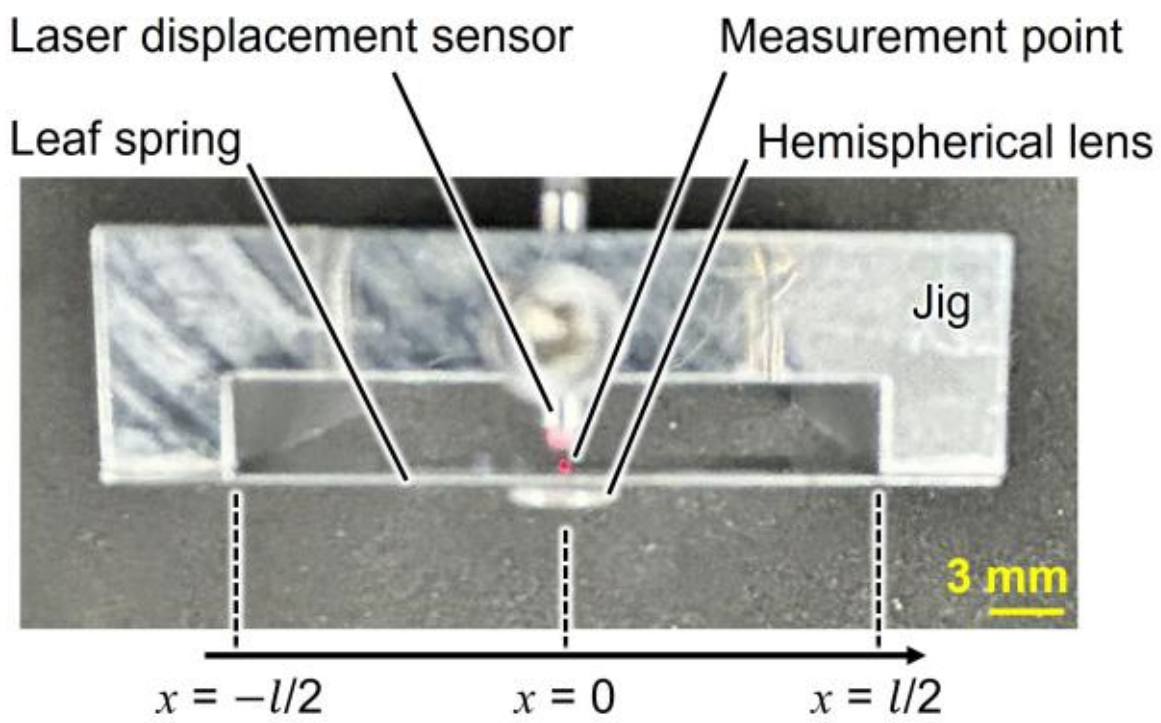

Figure S4. Overview of the squeeze-force measurement system. The origin (x = 0) corresponds to the center position of both the leaf spring and the hemispherical lens. The red dot indicates the position measured by the laser displacement sensor, at which the deflection of the leaf spring was measured.

5. Quantification of the Local Slip Length

Figures S5(a) and S5(b) show a typical fluorescence image of quantum dots acquired during gap narrowing and the corresponding PIV analysis result, respectively. Figure S5(c) shows the measured flow velocity $V(r)$ at center gap heights h_0 = 114 and 270 nm. An ImageJ plugin was used for the PIV analysis.[38] The interrogation window size was set to 24×24 pixels with 50% overlap. Since the pixel size was 0.27 μm/pix, the lateral resolution of the flow velocimetry was calculated to be 3.2 μm. The white symbols in Fig. S5(c) indicate the flow velocity averaged within $r \pm 2.5$ μm at each lateral position. The obtained average flow velocity values were in good agreement with the theoretical value $V_{\mathrm{ave}} = r(-dh/dt)/2h$, which is the z-directional average of the flow velocity profile based on Eq. (4). Over the center gap-height range of 99–293 nm, the velocity measurement error was estimated to be 3.5 μm/s based on the root-mean-square error between the experimental and theoretical average velocities. Because V_{ave} is independent of the interfacial velocities $V(0)$ and $V(h(r))$, this agreement demonstrates that the fluid velocity could be quantitatively measured from the translational velocity of the quantum dots. In contrast, at gap heights below 100 nm, the experimentally measured velocities deviated from the theoretical values. This deviation may be attributed to differences between the translational velocity of the quantum dots and the local fluid velocity arising from hydrodynamic interactions between the quantum dots and the solid surfaces or fluid slip at the quantum-dot surfaces. Therefore, in this study, the local slip length was evaluated over the center gap-height range of 99–293 nm, for which quantitative velocity measurements were experimentally validated.

The local slip lengths $b_1(r,h(r))$ and $b_2(r,h(r))$ were quantified by comparing the velocity distribution obtained by PIV with the theoretical velocity distribution calculated

from Eq. (4) at each lateral position and gap height. In the PIV analysis, fixed size interrogation windows were defined on the fluorescence image, and a single flow velocity value was calculated from the displacement of multiple fluorescent particles within each window. Therefore, the theoretical velocity distribution used for comparison needed to account for the number of fluorescent particles $n(h)$ present within a single interrogation window. To determine $n(h)$, fluorescence images were acquired during gap narrowing at a narrowing rate of 600 nm/s using the same sample as the one used for the flow velocity measurements. The number of quantum dots within a single interrogation window was then counted using the Analyze Particles function in ImageJ.[38] The number of quantum dots was measured to be in the range of 2–6 over a gap range of 100–300 nm (Fig. S6). A linear approximation of the experimental data gave the relationship between the number of quantum dots n and the gap h: $n = 0.016h + 1.3$.

Figure S5(d) shows the velocity distribution obtained from the velocity values within $r = 20 \pm 2.5$ μm in Fig. S5(c) ($h(r) = 127$ and 282 nm, corresponding to $h_0 = 114$ and 270 nm, respectively). Using the interfacial velocities $V(0)$ and $V(h(r))$ in Eq. (4) as fitting parameters, we performed fitting to minimize the root-mean-square error between the theoretical and experimental velocity distributions. For a gap of $h(r) = 282$ nm, no slip was observed. In contrast, for a smaller gap of $h(r) = 127$ nm, $V(0) = 17$ μm/s and $V(h(r)) = 20$ μm/s were obtained. Surface characterization showed that the stainless-steel-coated substrate was smoother and less wettable than the glass substrate. Surface roughness and wettability have been widely discussed in previous studies as factors affecting interfacial slip. In several solid–liquid systems, the slip length has been reported to increase with increasing contact angle, whereas surface roughness has been reported to suppress slip.[26,54] Based on these reported trends, $V(0) \leq V(h(r))$ was imposed as a constraint in

fitting the interfacial velocities in the present analysis. However, a recent study reported that the relationship between slip length and wettability does not necessarily follow this trend.[56] Therefore, the assumed relationship between the interfacial velocities does not represent a general relationship between the slip properties of the two interfaces, but rather serves as a constraint for the analysis of the present experimental system. From Eqs. (3) and (4), the slip length can then be expressed in terms of the interfacial velocity as follows:

$$b_1(r, h(r)) = \frac{V(0)}{3r(-dh/dt)/h(r)^2 - \left(4V(0) + 2V(h(r))\right)/h(r)},$$

$$b_2(r, h(r)) = \frac{V(h(r))}{3r(-dh/dt)/h(r)^2 - \left(2V(0) + 4V(h(r))\right)/h(r)} \tag{S4}$$

The local slip length at each lateral position r and gap height $h(r)$ was calculated using Eq. (S4).

The uncertainty in the fitted interfacial velocities $V(0)$ and $V(h(r))$ was evaluated based on the two-dimensional distribution of the root-mean-square error (RMSE) values obtained by comparing the experimental velocity distribution with the theoretical velocity distributions calculated for different combinations of $V(0)$ and $V(h(r))$. Figure S7 shows the RMSE distribution as a function of $V(0)$ and $V(h(r))$ at a gap height of $h(r)$ = 143 nm and a radial position of r = 20 μm. The cross indicates the fitted interfacial velocities $V(0)$ = 17.4 μm/s and $V(h(r))$ = 18.4 μm/s, at which the RMSE reached its minimum. The solid lines are contours connecting points with equal RMSE, and the percentages indicate the increase in RMSE relative to the minimum value at the fitted point. As shown in Fig. S7, the contours exhibit an elliptical shape elongated along a direction of negative slope. This indicates that an increase or decrease in one interfacial velocity can be partially compensated by a decrease or increase in the other, indicating some correlation between

the estimates of the two parameters. Nevertheless, Fig. S7 shows a distinct minimum-RMSE region, with the fitted point located near its center. The ranges of $V(0)$ and $V(h(r))$ within the region where the RMSE increased by 0.5% or less were approximately 3–4 µm/s. These values are comparable to the velocity measurement error (3.5 µm/s). Therefore, although $V(0)$ and $V(h(r))$ exhibit some correlation in the fitting, the coupling is not sufficiently strong to prevent the two interfacial velocities from being distinguished. These results indicate that the present method can determine the individual $V(0)$ and $V(h(r))$ with a resolution of several µm/s.

The measurement error in the slip length obtained by the present method was estimated as follows. The measurement errors in the gap height $h(r)$, gap-narrowing rate $-dh/dt$, and interfacial velocities $V(0)$ and $V(h(r))$ propagate into the slip length calculated using Eq. (S4). Therefore, the measurement error σ_b in the slip length can be expressed as follows:

$$\sigma_b = \sqrt{\left(\frac{\partial b}{\partial h(r)}\sigma_{h(r)}\right)^2 + \left(\frac{\partial b}{\partial(-dh/dt)}\sigma_{(-dh/dt)}\right)^2 + \left(\frac{\partial b}{\partial V(0)}\sigma_{V(0)}\right)^2 + \left(\frac{\partial b}{\partial V\big(h(r)\big)}\sigma_{V(h(r))}\right)^2} \quad \text{(S5)}$$

Here, $\sigma_{h(r)}$, $\sigma_{(-dh/dt)}$, $\sigma_{V(0)}$, and $\sigma_{V(h(r))}$ represent the measurement errors in the gap height, gap-narrowing rate, and interfacial velocities at the lower and upper surfaces, respectively. In the present study, $\sigma_{h(r)}$ and $\sigma_{(-dh/dt)}$ were 6 nm and 27 nm/s, respectively. The velocity measurement error of 3.5 µm/s was used for $\sigma_{V(0)}$ and $\sigma_{V(h(r))}$. Substitution of these values into Eq. (S5) yielded a measurement error in the slip length of 4 nm.

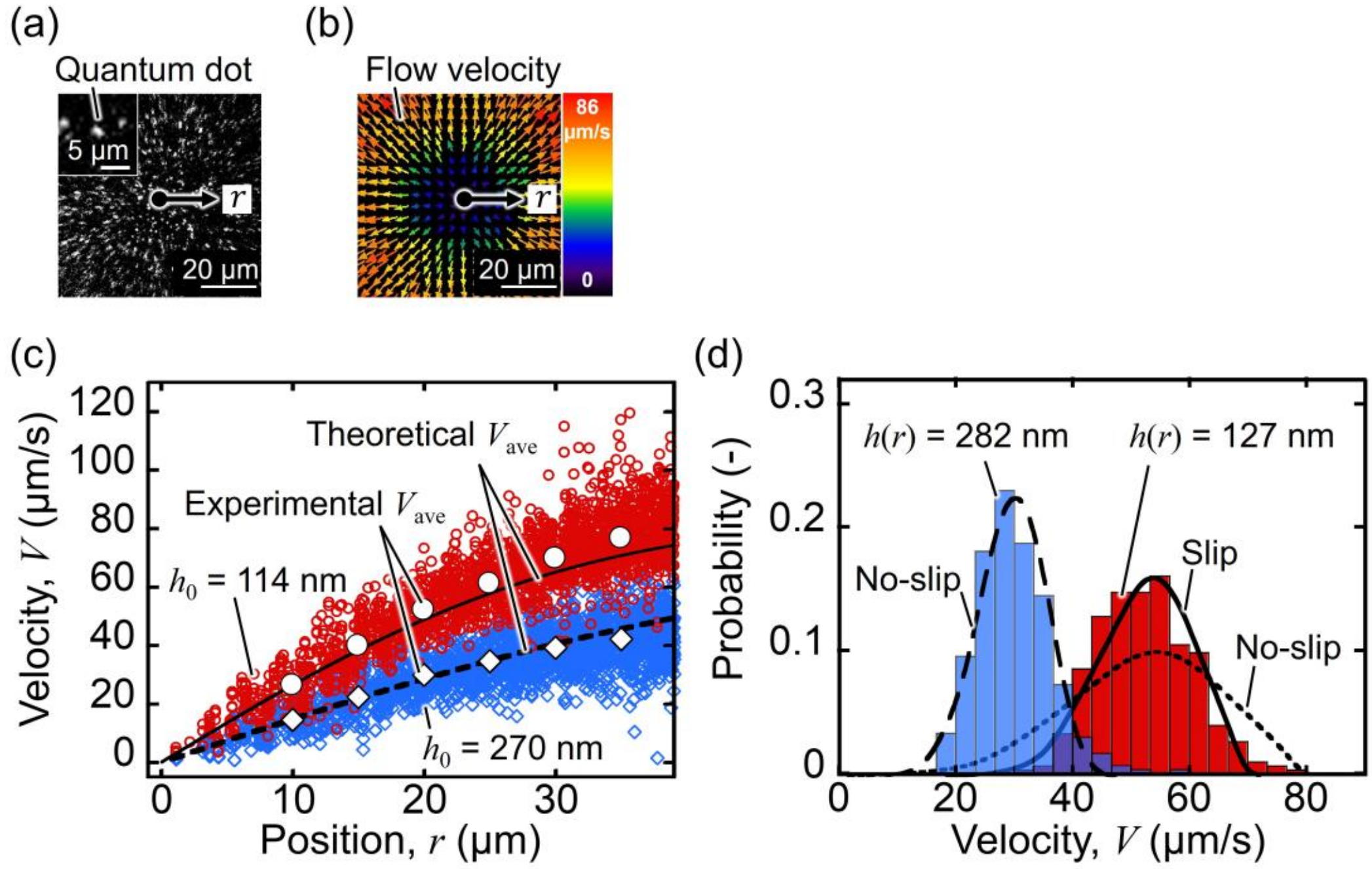

Figure S5. Quantification of local slip length. (a, b) Typical fluorescence image of quantum dots and the corresponding particle image velocimetry (PIV) result, respectively. (c) Measured flow velocity $V(r)$. Red and blue plots show the experimental results at h_0 = 114 and 270 nm, respectively. Filled circles and diamonds represent the averaged experimental values, and the solid and dashed curves represent the theoretical depth-averaged velocities, for h_0 = 114 and 270 nm, respectively. (d) Velocity distributions obtained by PIV. Red and blue bars show the experimental results at $h(r)$ = 127 and 282 nm, respectively. The solid curve represents the fitted theoretical result at $h(r)$ = 127 nm. The dotted curve represents the no-slip theoretical result at $h(r)$ = 127 nm, and the dashed curve represents the fitted no-slip result at $h(r)$ = 282 nm.

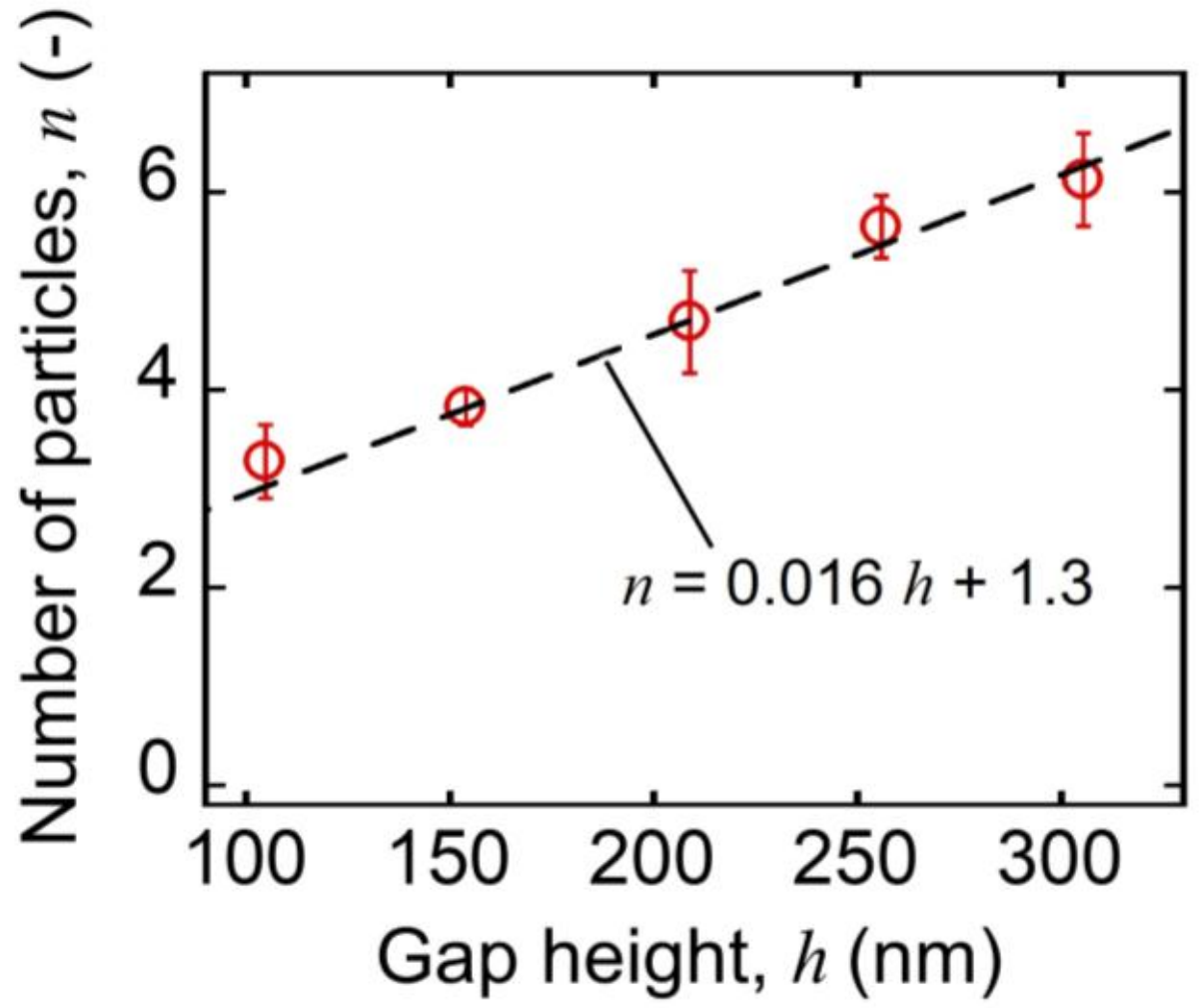

Figure S6. Relationship between the number of quantum dots contained in a single interrogation window for PIV analysis and the gap height *h*. Error bars show the standard deviation. The dashed line represents a linear fit.

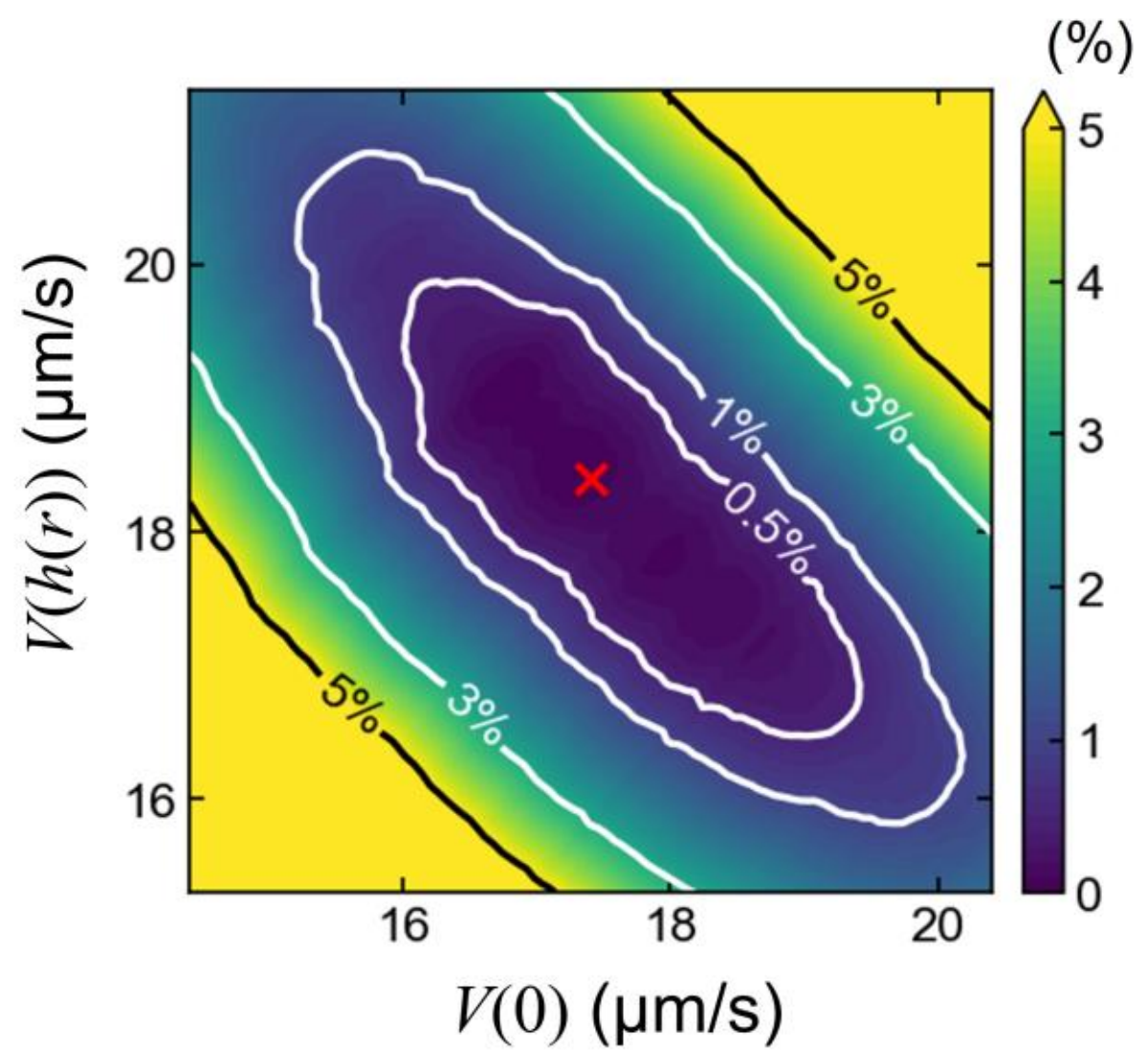

Figure S7. Distribution of the root-mean-square error (RMSE) values obtained by comparing the experimental velocity distribution with the theoretical velocity distributions at $h(r)$ = 143 nm and r = 20 μm. The cross indicates the fitted point ($V(0)$ = 17.4 μm/s and $V(h(r))$ = 18.4 μm/s), at which the RMSE reached its minimum. The color bar indicates the percentage increase in RMSE relative to the RMSE at the fitted point.

6. Relationship between Slip Length and Local Shear Rate at Different Gap Heights

Figure S8 shows the relationship between the slip length and apparent shear rate at the interface for different local gap heights $h(r)$. The measured slip lengths were grouped within a range of $h(r) \pm 25$ nm centered at each local gap height $h(r)$ and plotted separately. Since the local shear rate $\dot{\gamma}$ at the interface given by Eq. (2) is inversely proportional to $h(r)^2$, the data at smaller gap heights were distributed toward higher shear rates. Meanwhile, within the shear-rate ranges where data from different gap heights overlapped, the slip lengths were generally comparable, and no clear difference in the relationship between slip length and local shear rate was observed among the different gap heights. These results indicate that, over the gap-height range investigated in this study, the slip length did not exhibit a clear direct dependence on the gap height itself but instead showed a consistent relationship with the local shear rate at the interface.

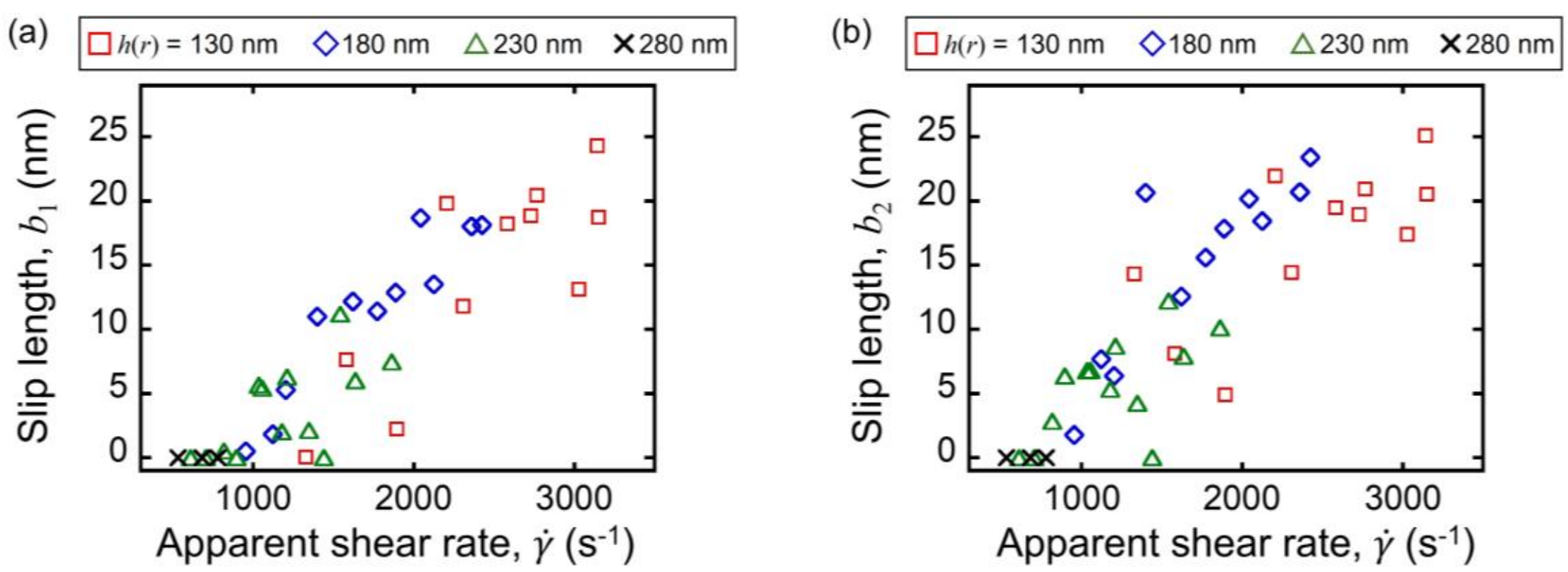

Figure S8. Relationship between slip length and apparent shear rate $\dot{\gamma}$ at the interface for different gap heights. (a) and (b) show the results for b_1 and b_2, respectively. The data were grouped within ranges of $h(r) \pm 25$ nm centered at each local gap height $h(r)$ and plotted separately.

7. Obtaining Constant Slip Length from Squeeze-Force Measurements

The constant slip length b_c was obtained based on the measured squeeze force shown in Fig. S9. For each actuator velocity U, b_c was determined by fitting Eq. (7) to the measured squeeze force F over the center gap height (h_0) range of 100–300 nm. The solid lines in Fig. S9 represent the squeeze force calculated from Eq. (7) using the obtained b_c.

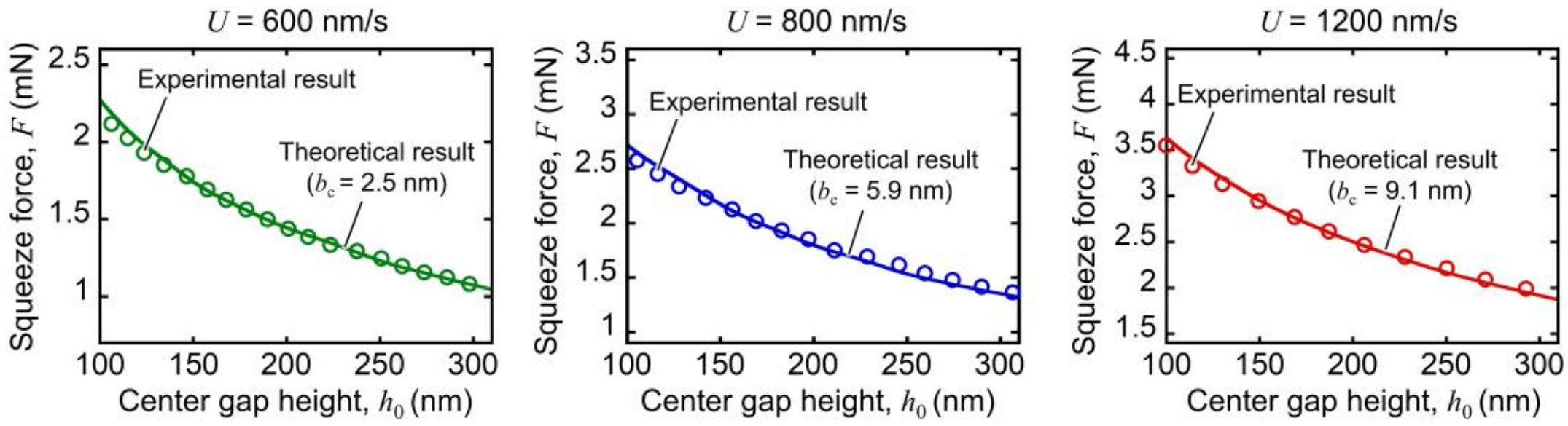

Figure S9. Results of the constant slip length obtained from the relationship between the squeeze force and the center gap height.

8. Gap-Height Dependence of Effective Viscosity

Based on the squeeze-force measurements obtained under conditions where interfacial slip could be assumed to be absent, we examined the applicability of the bulk viscosity over the gap-height range used for the squeeze-force measurements. The gap was narrowed at an actuator velocity of U = 100 nm/s, and the resulting squeeze force F was measured. At U = 100 nm/s and center gap heights of 100–300 nm, the apparent shear rate at the interface over the entire lateral region was lower than the critical shear rate for the onset of slip shown in Fig. 4(c). Therefore, the effect of interfacial slip was considered negligible under these conditions. The effective viscosity η_{eff} was then calculated using the following equation based on Eq. (S1):

$$\eta_{\mathrm{eff}} = \frac{F h_0}{6\pi R^2(-dh/dt)} \tag{S6}$$

Figure S10 shows the ratio of the effective viscosity to the bulk viscosity as a function of the center gap height. Over the center gap-height range of 100–300 nm, no clear variation in the effective viscosity beyond the scatter of the measured values was observed. These results indicate that the effective viscosity showed no clear gap-height dependence over the range investigated in this study.

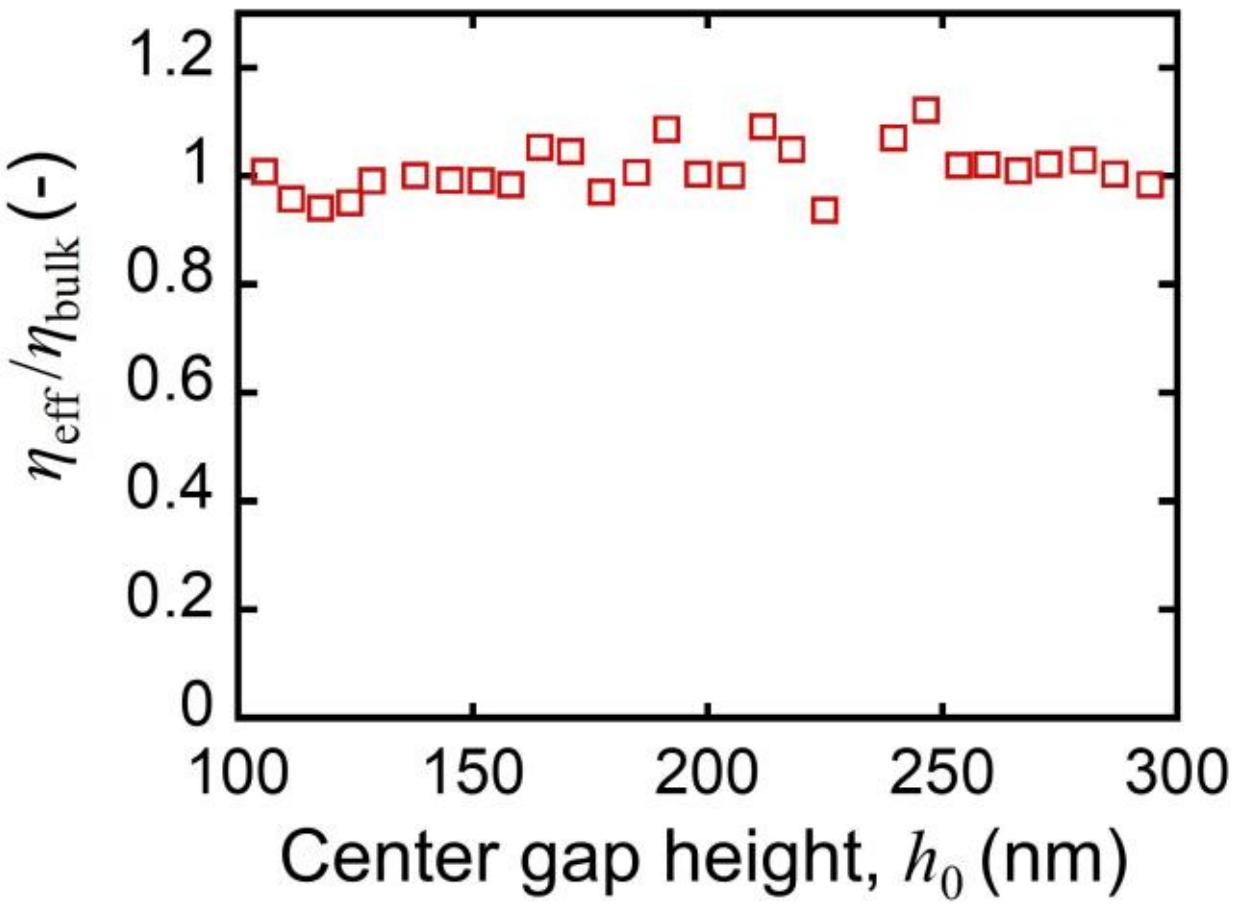

Figure S10. Ratio of the effective viscosity to the bulk viscosity as a function of the center gap height. The effective viscosity was calculated using Eq. (S6) based on the squeeze force measured at an actuator velocity of $U = 100$ nm/s.

9. Squeeze-Force Measurement Using a Sample without Quantum Dots

To examine the effect of quantum-dot addition on the slip characteristics, control squeeze-force measurements were performed using a sample without quantum dots. The sample used in this study was prepared by mixing a toluene dispersion of quantum dots with silicone oil. Therefore, to compare the samples with and without quantum dots, silicone oil containing the same amount of toluene but no quantum dots was used as the control sample. The piezoelectric actuator velocity U was set to 600 nm/s. Figure S11 compares the measured squeeze forces for the sample containing quantum dots and the control sample without quantum dots. The bulk viscosities of the samples with and without quantum dots were 127 and 121 mPa·s at 25 °C, respectively. Because the two samples had slightly different viscosities, the squeeze forces were normalized by the bulk

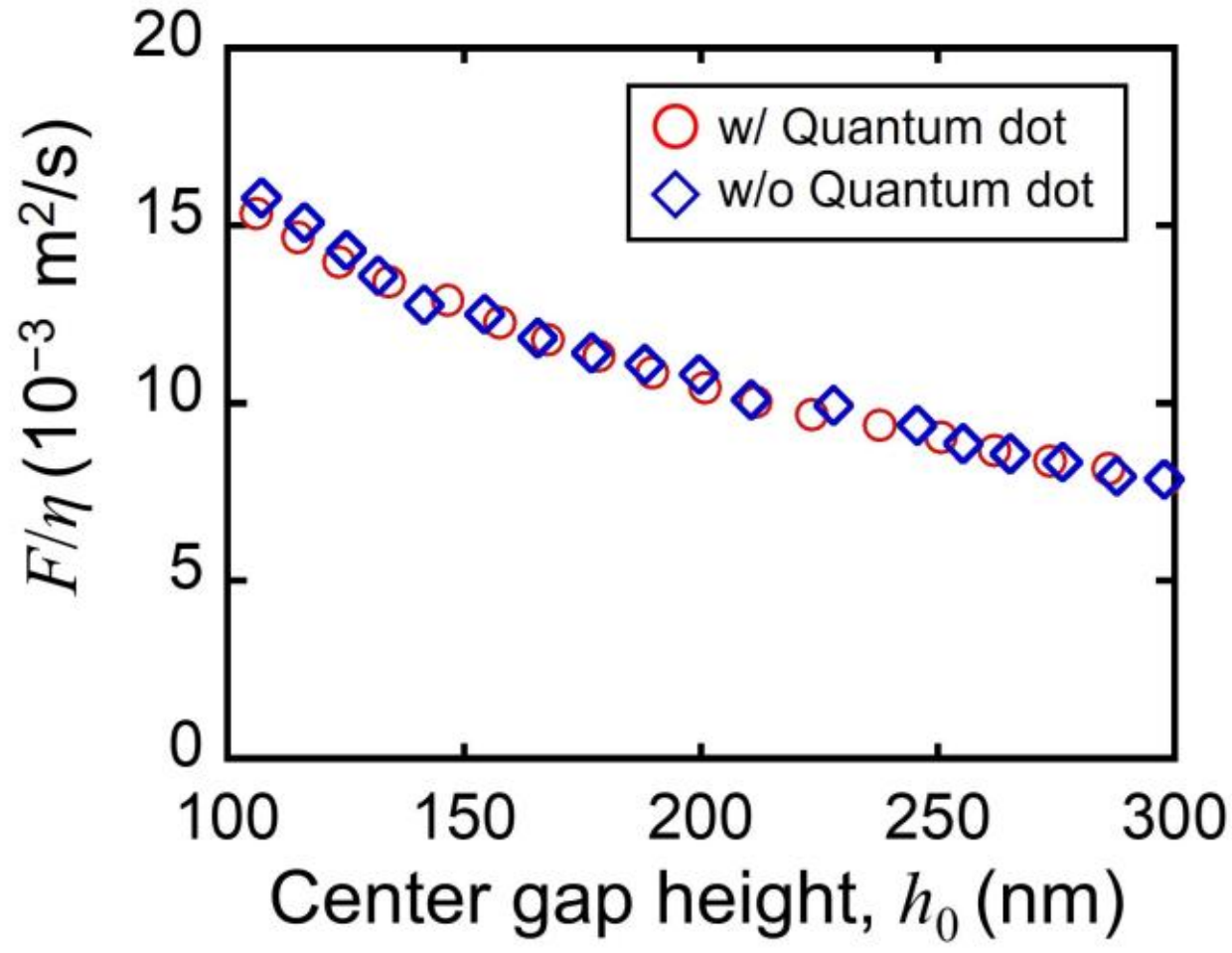

Figure S11. Squeeze-force measurements using samples with and without quantum dots at an actuator velocity of U = 600 nm/s. The squeeze force was normalized by the bulk viscosity of each sample to account for the difference in viscosity.

viscosity of each sample to account for the viscosity difference. No clear difference in the squeeze force was observed between the samples with and without quantum dots. Therefore, under the present experimental conditions, the addition of quantum dots was considered to have only a minor effect on the slip characteristics evaluated from the squeeze-force measurements.

REFERENCE

(S1) Craig, V. S.J.; Neto, C. In Situ Calibration of Colloid Probe Cantilevers in Force Microscopy: Hydrodynamic Drag on a Sphere Approaching a Wall. *Langmuir*. **2001**, *17*, 6018–6022.
